# Mitochondrial damage and impaired mitophagy contribute to disease progression in SCA6

**DOI:** 10.1007/s00401-023-02680-z

**Published:** 2024-01-29

**Authors:** Tsz Chui Sophia Leung, Eviatar Fields, Namrata Rana, Ru Yi Louisa Shen, Alexandra E. Bernstein, Anna A. Cook, Daniel E. Phillips, Alanna J. Watt

**Affiliations:** 1 Department of Biology, McGill University, Montreal, QC Canada; 2 Integrated Program in Neuroscience, McGill University, Montreal, QC Canada

**Keywords:** Transcriptome, Metabolomics, Mitochondria, Ataxia, Purkinje cell, Disease progression

## Abstract

Spinocerebellar ataxia type 6 (SCA6) is a neurodegenerative disease that manifests in midlife and progressively worsens with age. SCA6 is rare, and many patients are not diagnosed until long after disease onset. Whether disease-causing cellular alterations differ at different disease stages is currently unknown, but it is important to answer this question in order to identify appropriate therapeutic targets across disease duration. We used transcriptomics to identify changes in gene expression at disease onset in a well-established mouse model of SCA6 that recapitulates key disease features. We observed both up- and down-regulated genes with the major down-regulated gene ontology terms suggesting mitochondrial dysfunction. We explored mitochondrial function and structure and observed that changes in mitochondrial structure preceded changes in function, and that mitochondrial function was not significantly altered at disease onset but was impaired later during disease progression. We also detected elevated oxidative stress in cells at the same disease stage. In addition, we observed impairment in mitophagy that exacerbates mitochondrial dysfunction at late disease stages. In post-mortem SCA6 patient cerebellar tissue, we observed metabolic changes that are consistent with mitochondrial impairments, supporting our results from animal models being translatable to human disease. Our study reveals that mitochondrial dysfunction and impaired mitochondrial degradation likely contribute to disease progression in SCA6 and suggests that these could be promising targets for therapeutic interventions in particular for patients diagnosed after disease onset.

**Supplementary Information:**

The online version contains supplementary material available at 10.1007/s00401-023-02680-z.

## Introduction

Spinocerebellar ataxia type 6 (SCA6) is a neurodegenerative disease that typically first affects patients during midlife after which it progressively worsens with age [27, 66]. Post-mortem studies reveal extensive Purkinje cell death in the cerebellar vermis [26, 55, 78]. SCA6 is caused by CAG repeat expansion mutation in the *CACNA1A* gene that encodes both the α1A subunit of the P/Q voltage-dependent calcium channel [80], and a transcription factor, α1ACT [17]. Despite its likely role in transcriptional dysregulation, we have an incomplete understanding of the transcriptional changes that contribute to SCA6, limiting the development of effective treatments.

We utilize a mouse model of SCA6 that contains a hyper-expanded CAG repeat tract that recapitulates several features of human disease (SCA6^84Q/84Q^ mice, hereafter referred to as SCA6 mice): midlife onset, progressive motor impairment with age, and late Purkinje cell death [29, 76]. This allows us to examine cellular and molecular changes associated not only with disease onset, but also temporal alterations that are associated with disease progression. Given that SCA6 patients are often diagnosed post-onset [59, 60], it is important to understand the cellular pathophysiology that contributes to disease progression, since this may differ from mechanisms of disease onset.

To address this question, we used transcriptomics to explore the molecular changes underlying SCA6 at disease onset. We identified hundreds of significantly dysregulated genes, with both up- and down-regulated transcripts observed. Bioinformatic pathway analysis of these dysregulated genes revealed that the major families of downregulated genes were associated with impaired mitochondrial function and structure. Mitochondrial alterations have been implicated in many neurodegenerative diseases such as Alzheimer’s disease and Parkinson disease [5, 22], as well as other SCA subtypes SCA1 and SCA7 [62, 75], although mitochondrial dysfunction has not previously been observed in SCA6.

To determine how transcriptional changes affect mitochondrial function, we measured mitochondrial membrane potential in cerebellar Purkinje cells and found that it was normal at disease onset but impaired as disease progressed. In agreement with this, we observed an increase in oxidative stress in cerebellar Purkinje cells at later disease stages although not at disease onset. Intriguingly, these cellular dysfunctions were not limited to cerebellar Purkinje cells but was also observed in molecular layer interneurons, suggesting that other cerebellar cell types are also affected in SCA6, as has been demonstrated in other forms of ataxia [32, 49]. Next, we confirmed that mitochondrial structure is impaired in cerebellar Purkinje cells both at early and late disease stages using transmission electron microscopy. Since cells maintain mitochondrial quality in part by the elimination of damaged mitochondria through mitophagy, we explored whether mitophagy might be altered in SCA6. We observed that there was a reduction of markers of both autophagosomes and mitophagosomes, suggesting that the SCA6 cerebellum displays a progressive reduction in mitophagy that likely exacerbates mitochondrial dysfunction. To address whether our findings are representative of human disease, we analyzed post-mortem cerebellar tissues from SCA6 patients and non-neuropathological controls using metabolomics, and observed changes in metabolites that indicate mitochondrial dysfunction. Our findings uncover novel mitochondrial and mitophagy dysfunction that likely contributes to disease progression in SCA6 and highlight promising future avenues for therapeutic interventions for patients after disease onset.

## Materials and methods

### Animals

We used a knock-in mouse model of SCA6 containing a humanized 84 CAG repeat expansion mutation at the *Cacna1a* locus (SCA6^84Q/84Q^). To obtain homozygous SCA6 mice (SCA6^84Q/84Q^) and litter-matched WT control mice, we bred heterozygous mice (SCA6^84Q/+^) acquired from Jackson Laboratories (Bar Harbor, Maine; strain: B6.129S7-Cacna1atm3Hzo/J; stock number: 008683; RRID:IMSR_JAX:008683) [76]. Genotyping of the animals was performed using primer sequences provided by Jackson laboratories. Mice of both sexes were included in all experiments, and no animals were excluded from analysis. Breeding and animal procedures were carried out with the approval of the McGill Animal Care Committee in accordance with the Canadian Council on Animal Care guidelines.

### RNA extraction and RNA sequencing

The experimental area and equipment were cleaned with RNAseZAP (Sigma #R2020) to remove RNAse contamination. Mice were anesthetised with isoflurane until loss of toe pinch reflex, then decapitated and cerebellar vermis were dissected out, flash frozen on dry ice, and stored at − 80 °C until extraction. Cerebellar tissues were mechanically homogenized in TRIzol reagent (Invitrogen #15596026) by passing through 18-gauge then 23-gauge needles. Chloroform (Sigma #288306) was then added to the homogenate to separate total RNA into the aqueous phase, which was then recovered by precipitation with 70% ethanol. All procedures were performed on ice or at 4 °C. To further remove any organic carryover and DNA, RNA extracts were purified using RNeasy Mini Kit (Qiagen #74104) with DNase I treatment as described in manufacturer’s protocol.

RNA integrity was assessed with capillary electrophoresis (Agilent BioAnalyzer 2100) which generated an RNA integrity number (RIN). All samples have RIN over 9.3, indicating high RNA quality. Total RNA was sent to Genome Quebec for library preparation and sequencing. Library was created using NEB stranded mRNA library preparation, and sequencing was done on Illumina HiSeq 4000 generating 100 bp paired-end reads. These samples had a median of 53 million reads (interquartile range, 47–63 million reads), and a median of 92.5% mapping rate (interquartile range, 92–93%).

### RNA sequencing data analysis

FASTQ files were transferred to Compute Canada cloud server for processing. Reads were aligned to mouse reference genome (GRCm38) using HISAT2 version 2.1.0 [33]. Reads were quantified using HTSeq version 0.11. [1] in union mode. Differential expression analysis was done with DESeq2 version 1.24.0 in RStudio [37]. DESeq2 adjusted P values for multiple testing with a target ɑ = 0.05, and genes were considered DEGs at FDR < 0.05. For principal component analysis, normalized read data generated using DESeq2 was then log2 transformed. Enriched gene pathways in GO databases and KEGG pathway database were identified using gProfiler [50]. To identify significantly disrupted pathways, a limit of 1000 genes were used to filter out more general GO terms.

### Acute slice preparation and mitochondria membrane potential staining

Slice preparation was performed as previously described [14]. Mice were deeply anesthetised with intraperitoneal injection of 2,2,2-tribromoethanol (Avertin) until the loss of toe pinch reflex, followed by an intracardiac perfusion with approximately 30 ml of ice-cold partial sucrose replacement slicing solution (111 mM sucrose, 25 mM glucose, 50 mM NaCl, 2.5 mM KCl, 1.25 mM NaH_2_PO_4_, 25 mM NaHCO_3_, 0.65 mM CaCl_2_ and 10 mM MgCl_2_, bubbled with 95% O_2_ and 5% CO_2_ to maintain pH at 7.3). Mice were then rapidly decapitated, and the brain was dissected out, which was then sectioned into 150 µm thick slices using a VT 1200S vibratome (Leica Microsystems, Wetzlar, Germany). Slices were incubated in artificial cerebrospinal fluid (ACSF: 20 mM glucose, 125 mM NaCl, 2.5 mM KCl, 1.25 mM NaH_2_PO_4_, 25 mM NaHCO_3_, 2 mM CaCl_2_ and 1 mM MgCl_2_, bubbled with 95% O_2_ and 5% CO_2_ to maintain pH at 7.3; osmolality ~ 320 mOsm) at 37°C for 45 min for recovery.

To stain slices with Tetramethylrhodamine, Ethyl Ester, Perchlorate (TMRE, Invitrogen #T669), slices were transferred to a 24-well plate, one slice per well, and incubated in 10 nM of TMRE (prepared fresh the day of experiment by serial dilution with ACSF from 200 µM stock in DMSO) at 37°C for 30 min in dark. As a control, 40 µM of carbonyl cyanide-p-trifluoromethoxyphenylhydrazone (FCCP) was added to collapse membrane potential which prevents TMRE accumulation in mitochondria. During image acquisition, slices were left in 10 nM TMRE to prevent recalibration of the dye. Using an LSM800 confocal microscope (Zeiss) and excitation at 561 nm, z-stack images were acquired from 4 to 6 slices/animal. During each experimental session, cerebella from two animals, one of each genotype, were used to manage uncontrolled variables. Blinding was achieved by pre-selecting the animal pairs for each session and referring to each animal only by their IDs.

Image analysis was performed in Fiji (ImageJ; US National Institutes of Health) [52, 57]. Z-projection of 11 optical slices (slice interval at 0.5 µm, total section of 5 µm) max intensity was used to measure signal intensity within regions of interest. Data were normalized to WT data from the same experimental session.

### Immunohistochemistry

Tissue sections for immunohistochemistry was prepared as previously described [14]. Anesthesia of mice were induced and maintained with isoflurane through inhalation, confirmed with absence of toe pinch reflex. Then, intracardiac perfusion was performed with a flush of ice-cold phosphate-buffered saline (PBS; 0.1 M, pH 7.4) with heparin salt (5.6 μg/ml), followed by 40 ml of 4% paraformaldehyde (PFA) in phosphate buffer (pH 7.4), both at the rate of 50 ml/min. Brain was rapidly removed and incubated in 4% PFA at 4°C overnight on an orbital shaker at 70 rpm before being transferred to PBS with 0.05% sodium azide for short term storage at 4°C. The cerebellum was then sliced using a vibrating microtome (5100mz Vibratome, Campden Instruments, Loughborough, UK) into sections of 80 or 100 µm thickness. Slices were collected serially and were suspended in 24-well plate containing PBS with 0.05% sodium azide.

For each experiment, slices from all animals were stained simultaneously in one setting, and master mixes of antibodies were made to minimize batch effects. All incubation steps were done on an orbital shaker at 70 rpm to achieve even staining. Free-floating slices were first rinsed with washing solution (PBS with 0.4% Triton X-100), then incubated in primary antibody (Supplementary Table 2) and blocking solution (PBS, 0.4% Triton X-100, 5% Bovine serum albumin (BSA), 0.05% sodium azide) for 3 days at room temperature. Slices were then rinsed in washing solution and incubated for 90 min with secondary antibodies (Table 2). For experiments involving antibodies derived from mouse, an additional 30-min incubation with Anti-Mouse IgG Fab fragments was added prior to secondary antibody incubation. After a final washing solution rinse, stained slices were mounted using ProLong Gold Antifade mounting medium (Thermo Fisher Scientific, Waltham, USA) and stored in dark at 4°C prior to imaging.

### Image acquisition and analysis

Images were acquired using an LSM800 confocal microscope (Zeiss) on Zen Blue software. For analyses that detect signal intensity within the entire Purkinje cell body area (Figs. 3, 4 and 6e), images were acquired using a 20 × objective at 1024 × 1024-pixel resolution. For analyses concerning subcellular structures (Fig. 6a–d), images were acquired using a 40 × objective at 2048 × 2048-pixel resolution. Laser settings were kept identical for each experiment. All imaging and analysis were performed in lobule III of the anterior vermis, a region of the cerebellum where cellular deficits in SCA6 were shown [14, 29]. Both image acquisition and image analysis were carried out blinded to the experimental conditions.

Image analysis was done in Fiji [52, 57]. For Figs. 3, 4 and 6e, Purkinje cell and molecular layer interneurons bodies were delineated by hand using calbindin and parvalbumin as markers respectively, which were then overlaid onto regions of interest to measure signal intensity within these cells. For Fig. 6a–d, object-based colocalization were used. Images were pre-processed with *Background Subtraction*, then an auto-threshold protocols was applied to segment lysosome (stained by Lamp1, using *RenyiEntropy*) and mitochondria (stained by CoxIV, using *Moments*) from the background. Then, the area of the intersection of lysosome and mitochondria was calculated using the AND operator in *Image Calculator.*

To generate representative images, linear adjustments of the brightness and contrast were applied to improve the legibility. Identical adjustments were applied to all images within a data set.

### Transmission electron microscopy

For tissue fixation, mouse anesthesia was induced and maintained with isoflurane through inhalation, confirmed with absence of toe pinch reflex. Then, intracardiac perfusion was performed with a flush of ice-cold phosphate-buffered saline (PBS; 0.1 M, pH 7.4) with heparin salt (5.6 μg/ml), followed by 40 ml of 2% paraformaldehyde (Electron Microscopy Sciences) and 2% glutaraldehyde (Electron Microscopy Sciences) in 0.1 M phosphate buffer in (pH 7.4), both at the rate of 50 ml/min. Brain was rapidly removed and incubated in 2.5% glutaraldehyde in 0.1 M sodium cacodylate (Electron Microscopy Sciences) at 4°C for one week on an orbital shaker at 70 rpm. Lobule III of the cerebellar vermis were micro-dissected out under light microscope and sent for dehydration, embedding, sectioning, and staining processed by Facility for Electron Microscopy Research (FEMR) at McGill University, as described before [35].

Images were acquired using FEI Tecnai G2 Spirit Twin Cryo-TEM (FEI Company, Hillsboro, OR, USA) at 120 kV and visualized with an AMT XR80C 8-megapixel CCD camera (Advanced Microscopy Techniques, Woburn, MA, USA) at 1900 × and 4800 × resolutions. Only one cross section image was taken for each Purkinje cell body, thus each Purkinje cell is analysed only once. EM images from each timepoint were analysed in parallel by two independent analysts, who were blinded to genotypes. They identified and counted all mitochondria in the Purkinje cell body and annotated them individually as healthy or damaged based on the cristae content and the integrity of mitochondrial membrane. Data from one observer is shown and data from the other observer is provided in the supplementary material (Supplementary Fig. 4c–f).

### Accelerating rotarod

We used an accelerating paradigm on a rotarod (Stoelting, IITC) as previously described [14, 28, 29], which robustly detects motor coordination deficits in SCA6 mice. Mice were allowed to acclimatize in the experimental room for 1 h prior to testing. Mice were then placed on rotating rods, which accelerates from 4 to 40 rpm over 5 min and stays at 40 rpm for another 5 min. Latency to fall off the rod was recorded. If a mouse fell off within 5 s, it was considered as an invalid trial and the animal was immediately retested. Mice performed the assay four times per day with 10 min rest between trials, for five consecutive days. Average latencies of each day were shown.

### Human post-mortem cerebellar tissues

SCA6 patient’s post-mortem cerebellar tissues were donated by Dr. Arnulf Koeppen (Albany Stratton Veterans Affairs Medical Center, Albany, New York, USA). Age- and sex- matched non-neuropathological control cerebellar tissues were obtained from The Douglas Bell Canada Brain Bank. Tissues were shipped in pellet dry ice and stored at − 80 °C upon arrival.

### Tissue extraction and LC–MS/MS

Post-mortem human cerebellar tissues were crushed to fine powder in liquid nitrogen using a pre-chilled mortar and pestle. Approximately 19 mg of crushed tissue (19.2 ± 0.2 mg) were quickly weighed out and transferred to tubes stored in dry ice to minimise tissue thawing. Tissues were then extracted with 31.6% methanol (Fisher Scientific, Ottawa, Ontario)/36.7% acetonitrile (Fisher Scientific, Ottawa, Ontario) in LC/MS grade water containing 1 mg/ml of N-ethylmaleimide (Sigma-Aldrich, Oakville, Ontario) to protected free thiols [54]. Then, tissues were lysed and homogenized by bead-beating for 2 min at 30 Hz using 4 ceramic beads (2 mm) per sample (SpeedMill Plus, Jena Analytik). Tissue extracts were partitioned into an aqueous layer, a protein intermediate layer and an organic layer following dichloromethane treatment and centrifugation. The protein layers were dried by vacuum centrifugation and analyzed for total protein concentration using Bradford assay. The aqueous supernatants were dried by vacuum centrifugation with sample temperature maintained at −4°C (Labconco, Kansas City MO, USA). Dried extracts were subsequently re-suspended in 50 μL of chilled H2O and clarified by centrifugation at 1°C.

#### Ion pairing

For targeted metabolite analysis, samples were injected onto an Agilent 6470 Triple Quadrupole (QQQ)–LC–MS/MS (Agilent Technologies). Chromatographic separation of metabolites was achieved by using a 1290 Infinity ultra-performance quaternary pump liquid chromatography system (Agilent Technologies). The mass spectrometer was equipped with a Jet StreamTM electrospray ionization source, and samples were analyzed in negative mode. The source-gas temperature and flow were set at 150°C and 13  L/min, respectively, the nebulizer pressure was set at 45 psi, and capillary voltage was set at 2000 V. Multiple reaction monitoring parameters (qualifier/quantifier ions and retention times) were obtained and optimized using authentic metabolite standards (Sigma-Aldrich, Oakville, Ontario).

Chromatographic separation of the isomers and other metabolites was achieved by using a Zorbax Extend C18 column 1.8 μm, 2.1 × 150 mm^2^ with guard column 1.8 μm, 2.1 × 5 mm^2^ (Agilent Technologies). The chromatographic gradient started at 100% mobile phase A (97% water, 3% methanol, 10 mM tributylamine, 15 mM acetic acid, 5 µM medronic acid) for 2.5 min, followed with a 5-min gradient to 20% mobile phase C (methanol, 10 mM tributylamine, 15 mM acetic acid, 5 µM medronic acid), a 5.5-min gradient to 45% C and a 7-min gradient to 99% C at a flow rate of 0.25 mL/min. This was followed by a 4-min hold time at 100% mobile phase C. The column was restored by back-washing with 99% mobile phase D (90% ACN) for 3 min at 0.25 mL/min, followed by increase of the flow rate to 0.8 mL/min over 0.5 min and a 3.85-min hold, after which the flow rate was decreased to 0.6 mL/min over 0.15 min. The column was then re-equilibrated at 100% A over 0.75 min, during which the flow rate was decreased to 0.4 mL/min, and held for 7.65 min. One minute before the next injection, the flow was brought back to forward flow at 0.25 mL/min. For all LC–MS analyses, 5  μL of sample was injected. The column temperature was maintained at 35°C.

#### Amino acids and derivatives

Since the above ion pairing method is limited to only negative ionization, amino acids and other related metabolites were collected using an Agilent 6470 Triple Quadrupole (QQQ)-LC–MS/MS. Chromatography was achieved using a 1290 Infinity ultra-performance LC system (Agilent Technologies, Santa Clara, CA, USA). Mass spectrometer was equipped with a Jet Stream electrospray ionization (ESI) source and samples were analyzed in positive mode. Multiple reaction monitoring was optimized, and retention times confirmed on authentic metabolite standards. Source gas temperature and flow were set at 300°C and 5 L/min respectively, nebulizer pressure was set at 45 psi and capillary voltage was set at 3500 V. The autosampler temperature was maintained at 4 °C.

Chromatographic separation of creatine was achieved using an Intrada Amino Acid column 3 μm, 3.0 × 150 mm (Imtakt Corp, JAPAN). The chromatographic gradient started at 100% mobile phase B (0.3% formic acid in ACN) with a 3 min gradient to 27% mobile phase A (100 mM ammonium formate in 20% ACN / 80% water) followed with a 19.5 min gradient to 100% A at a flow rate of 0.6 ml/min. This was followed by a 5.5 min hold time at 100% mobile phase A and a subsequent re-equilibration time (7 min) before next injection. The column temperature was maintained at 10°C. Relative concentrations were determined from external calibration curves of standards dissolved in water.

All LC–MS/MS data were analyzed using MassHunter Quant (Agilent Technologies). No additional corrections were made for ion suppression; thus, concentrations are relative to the calibration curve and not absolute. Relative concentration of each compound was normalized to total protein amount and data are represented as fold change relative to the average of control.

### Statistics

Data were first tested for normalcy and statistical comparisons were performed using Student’s *t* test or Mann–Whitney *U* test using JMP software (SAS, Cary, NC). Data are reported as box plots, where the box represents 1st quartile, median and 3rd quartile, and the whiskers represent one standard deviation from median.

## Results

### Transcriptional alterations in SCA6 cerebellum

**Fig. 1:**
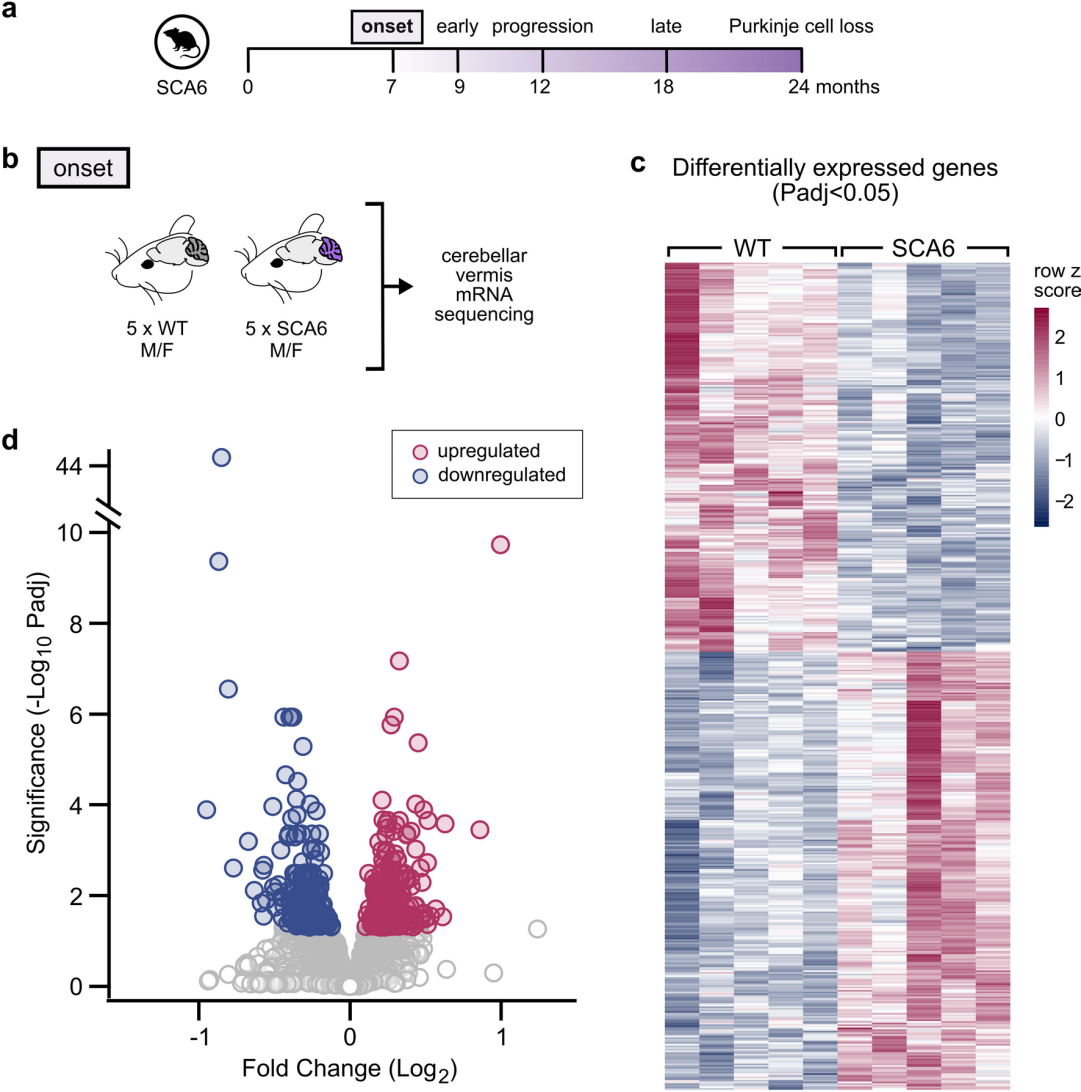
Gene expression changes in SCA6 cerebellum at disease onset. (**a**) Timeline of disease progression in SCA6 mouse model. (**b**) Cerebellar vermis tissue was extracted from male and female WT and SCA6 mice (N = 5 per genotype) for sequencing. (**c**) RNA sequencing revealed 513 DEGs in SCA6 compared to WT (Padj < 0.05). Each column of the heatmap represents an animal, each row represents a gene. Refer to Supplementary Table 1 for complete list of genes. (**d**) Volcano plot shows the distribution of fold changes of the disrupted genes in SCA6. Colored markers represent significant genes, grey markers are non-significantly changed genes

To identify novel molecular targets that may contribute to SCA6 disease, we profiled gene expression changes using RNA sequencing (RNA-seq) in a knock-in mouse model of SCA6 [76]. We compared SCA6 and litter-matched wildtype (WT) control mouse cerebellar vermis at disease onset (7 months), an age when motor coordination deficits start to be observed but prior to Purkinje cell degeneration (Fig. 1a) [29]. Animals were selected based on rotarod performance to confirm that SCA6 mice included in this analysis displayed impaired motor coordination (Supplementary Fig. 1a), with both sexes included. We micro-dissected the cerebellar vermis from SCA6 and WT mice (N = 5 per group; Fig. 1b), then extracted and sequenced the total mRNA from tissue homogenates. To gain insight into sources of variability among samples, we performed principal component analysis (PCA) on the RNA-seq data. PC1 (33.4%) and PC2 (24.5%) together accounted for slightly over half of the variability in the dataset (Supplementary Fig. 1b), suggesting that there could be more than two main sources of variability. The samples were loosely grouped into two clusters corresponding to the two genotypes (Supplementary Fig. 1c), suggesting that despite the appreciable level of variability, the expression profile of SCA6 animals was distinguishable from that of WT animals. Interestingly, however, data did not cluster based on sex, suggesting sex difference was not a main source of variability in transcription profile (Supplementary Fig. 1c).

To gain insight into disrupted molecular signatures, we next performed differential expression analysis, and found that there were 513 differentially expressed genes (DEGs) that showed significant differences in SCA6 mice (Padj < 0.05; Fig. 1c and Supplementary Table 1). Of these, ~ half (54%) were upregulated, and ~ half (46%) were downregulated (Fig. 1c). This suggests that widespread transcriptional dysregulation is present in SCA6, consistent with the mutated gene, *Cacna1a,* encoding a transcription factor [17]. The majority of dysregulated genes were altered by only moderate fold changes from WT level, with most genes dysregulated to between 50 and 150%, or − 0.585 and 0.585 on log2 scale (Fig. 1d). Of these more than 500 DEGs, certain genes displayed changes as predicted: e.g., *Cacna1a* had reduced expression level in SCA6 than WT (fold change = 0.791; Padj = 0.0152; Supplementary Fig. 1d), suggesting that the CAG repeat expansion mutation decreases expression of both the P/Q calcium channel and the transcription factor that it encodes (consistent with [76]). However, the function of many of the most highly dysregulated genes in the cerebellum is largely unknown (see Supplementary Table 1).

**Fig. 2:**
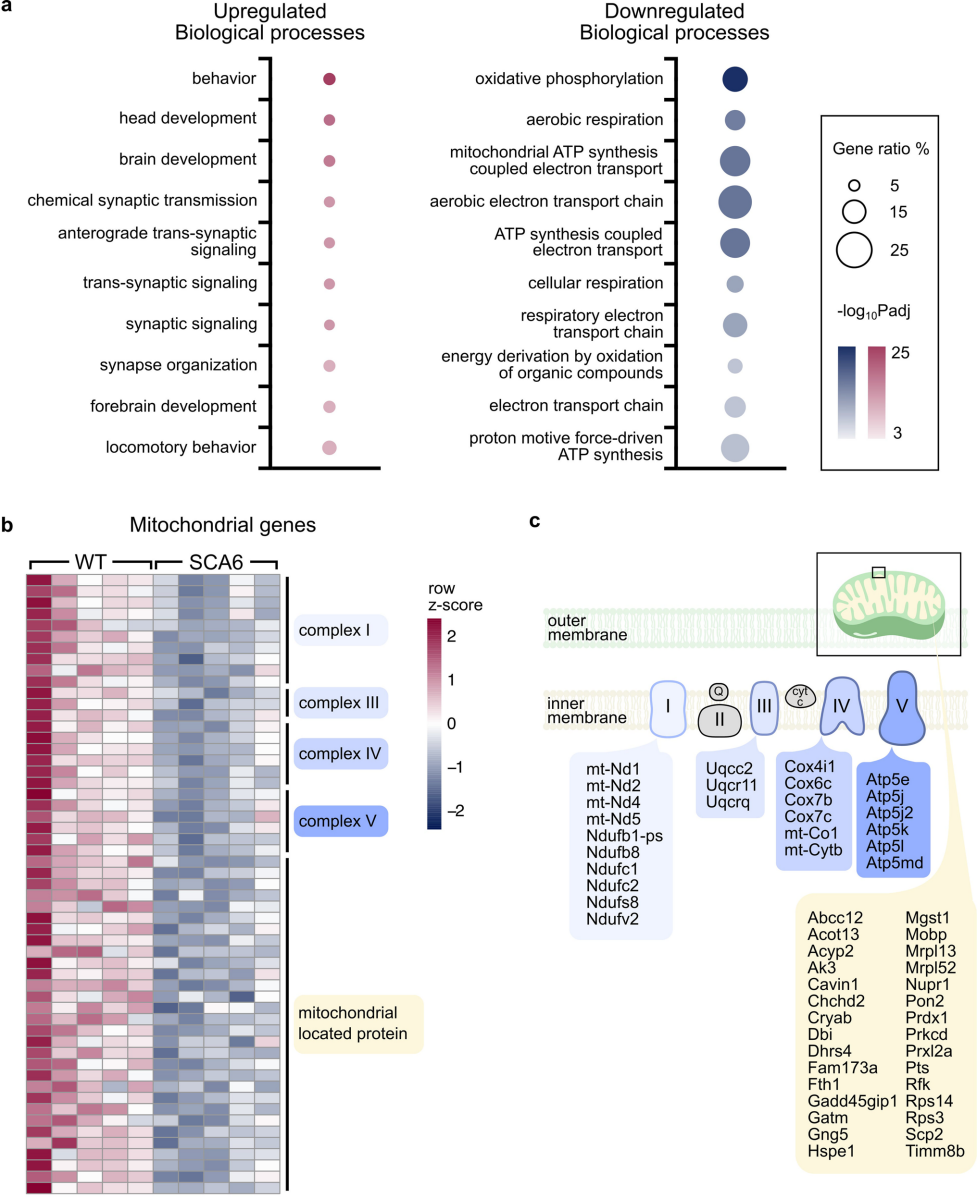
Mitochondrial genes are downregulated in SCA6. (**a**) GO term enrichment analysis revealed the top biological processes that are up- (left) and down- (right) regulated. The proportion of dysregulated genes per GO term is larger for down-regulated compared to up-regulated GO terms (gene ratio, indicated by size of the circle). (**b**) Heatmap showing down-regulated mitochondrial-related genes grouped by location within the mitochondria showed that these changes are consistent in same direction in all 5 SCA6 animals. (**c**) Genes encoding complexes of the electron transport chain and mitochondria membrane were downregulated

To interpret the list of DEGs in functional terms, we performed gene ontology (GO) term enrichment analysis on the list of DEGs to identify impacted biological processes, rather than investigating single genes or proteins. We mapped both up- and down-regulated genes onto biological process GO terms and detected the statistically over-represented terms. We found that the gene ratios (the number of mapped DEGs/the number of genes associated with the GO term) were higher for the downregulated terms compared to the upregulated terms (Fig. 2a, indicated by the size of the circles), suggesting that these downregulated biological processes may be more severely affected. Among the significantly upregulated GO terms, we found that the top terms were those involved in behavior, development, and synapse organization and signaling (Fig. 2a). This is interesting since synaptic dysregulation has been observed in SCA6 models [41], and developmental alterations have been observed previously in the same mouse model [30]. Since the RNA-seq was performed at a mid-life timepoint, our data suggest that either the gene expression changes that induce developmental deficits persist into adulthood, or the development GO terms upregulated in SCA6 may lack temporal specificity.

### Mitochondrial genes are downregulated in SCA6

In contrast to the top upregulated GO terms, the top downregulated GO terms (Fig. 2a) included larger gene ratios that were significantly over-represented compared to the upregulated GO terms (indicated by larger and darker markers). Interestingly, these downregulated GO terms correspond to biological processes that have not previously been implicated in SCA6 that strongly suggest mitochondrial dysfunction. Genes mapped to these GO terms include both genomic (e.g., Atp5e) and mitochondrial (e.g., mt-Cytb) genes and include genes that encode for complex subunits in the electron transport chain (ETC) (Fig. 2b, c). These genes were consistently downregulated in all 5 SCA6 RNA-seq samples (Fig. 2b). This suggests that there could be a reduction of mitochondria biogenesis, a global reduction of the pool size of mitochondria, and/or a disruption to mitochondrial respiratory capacity in SCA6. Since mitochondrial dysfunction is a novel impairment that has not previously been implicated in SCA6 disease pathology, we chose to study it further.

### Lowered mitochondrial membrane potential as SCA6 progresses

**Fig. 3:**
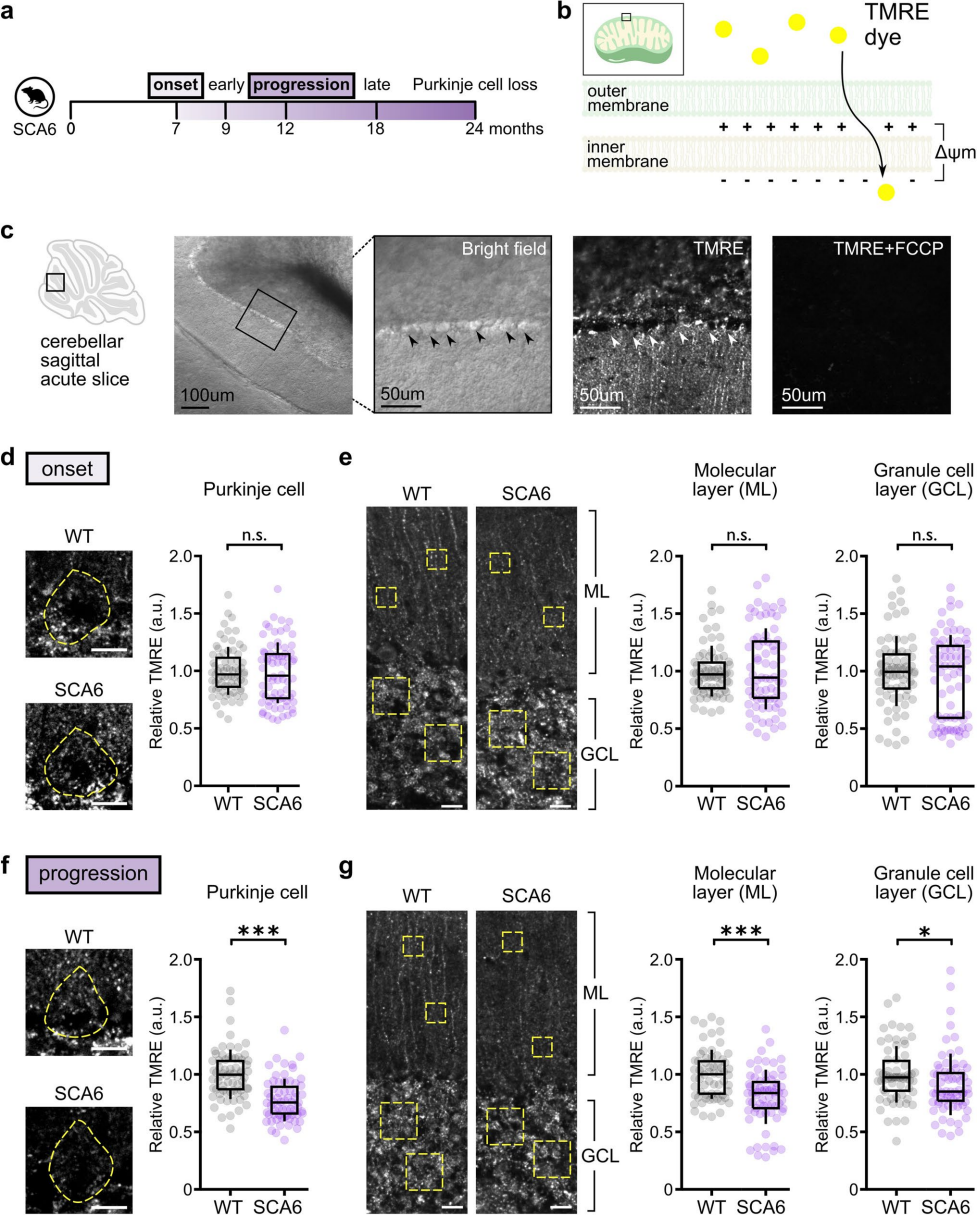
SCA6 cerebellar mitochondria have a reduced membrane potential (Δψ_m_), resulting in impaired cellular respiration as the disease progresses. (**a**) Δψ_m_ was assayed at disease onset and disease progression. (**b**) The fluorescent dye TMRE accumulates in mitochondria in proportion to Δψ_m_. (**c**) Purkinje cell bodies could be identified by their location between the molecular layer and granule cell layer under bright field and laser-scanning confocal microscopy. FCCP collapses mitochondrial Δψ_m_ and reduced TMRE signal to background levels as a negative control. (**d**, **e**) Data from disease onset (7 months). (**d**) Representative TMRE images of Purkinje cell bodies (outlined) in WT and SCA6. Relative TMRE signals showed no difference between genotypes (WT: n = 74 cells from N = 3 mice; SCA6: n = 74 cells from N = 3 mice; not significantly different, P = 0.617) (**e**) Representative TMRE images of a section of the cerebellar sagittal slice showing molecular and granule cell layer. Yellow dotted boxes illustrate regions of interest (ROIs). There were no differences in relative TMRE signal between genotypes at disease onset in either the molecular (WT: n = 74 ROI from N = 3 mice; SCA6: n = 73 ROI from N = 3 mice; P = 0.802) or the granule cell layer (WT: n = 74 ROI from N = 3 mice; SCA6: n = 73 ROI from N = 3 mice; P = 0.795) (**f**, **g**) Data from a later timepoint as SCA6 progresses (12 months; progression). (**f**) Representative TMRE images of Purkinje cell bodies (outlined) in WT and SCA6. At this later age, TMRE signals were significantly reduced in SCA6 (WT: n = 68 cells from N = 3 mice; SCA6: n = 65 cells from N = 3 mice, P < 0.0001) (**g**) Representative TMRE images of a section of the cerebellar sagittal slice showing molecular layer and granule cell layer. Relative TMRE signal was reduced in SCA6 in both molecular layer (WT: n = 64 ROI from N = 3 mice; SCA6: n = 64 ROI from N = 3 mice; P < 0.0001) and granule cell layer (WT: n = 64 ROI from N = 3 mice; SCA6: n = 68 ROI from N = 3 mice; P < 0.05). Scale bar for (**d**)–(**g**) = 20 µm. Mann Whitney *U* test was used for all statistical comparisons. * P < 0.05, *** P < 0.0001, n.s. P > 0.05

Our RNA-seq data demonstrated reduced expression of ETC genes in SCA6 cerebellar vermis. Since ETC complexes are required to sustain mitochondrial respiration, we hypothesized that this may result in a lower respiratory capacity of SCA6 mitochondria. To test this, we examined the mitochondrial membrane potential (Δψ_m_) in individual neurons within live cerebellar tissue at two disease stages: disease onset (7 months) and disease progression (12 months; Fig. 3a). Δψ_m_ is generated by complexes in the ETC and, together with the mitochondrial pH gradient, it provides the bioenergetic driving force for respiration, or ATP production [81]. We loaded live acute cerebellar slices from WT and SCA6 animals with 10 nM of Tetramethylrhodamine, Ethyl Ester (TMRE), a fluorescent dye that accumulates within mitochondria in proportion to their Δψ_m_ (Fig. 3b), and then imaged the slices on a laser scanning confocal microscope to determine TMRE intensity within Purkinje cells. Purkinje cell bodies could be reliably identified based on their size and location between the molecular layer and granule cell layer under both bright field and laser microscopy (Fig. 3c). As a control, carbonyl cyanide-p-trifluoromethoxyphenylhydrazone (FCCP) was administered to collapse Δψ_m_ which reduced the TMRE signal to background level, confirming that the fluorescent signal indeed came from TMRE accumulated in mitochondria (Fig. 3c).

We collected TMRE images from the same location in the cerebellum, the anterior lobule, and from both genotypes in each experimental session to minimize technical varibility (N = 3 animals per genotype, n = 16–26 cells per animal for each timepoint). TMRE signals were punctate, resembling the distribution of mitochondria in cells (Fig. 3d–g), and were observed across the cerebellar cortical layers (Fig. 3c). Thus, although SCA6 pathology has been thought to be primarily expressed in cerebellar Purkinje cells [25, 70], we had the opportunity to examine changes in Δψ_m_ in mitochondria within each cortical layer: we visually identified Purkinje cell bodies within the Purkinje cell layer (Fig. 3d, f), and also measured Δψ_m_ in the molecular layer, likely arising from a heterogenous population of mitochondria located in Purkinje cell dendrites, parallel fiber, molecular layer interneurons, and Bergmann glia; as well as in the granule cell layer, likely representing a mixed population of mitochondria located in granule cells, Golgi cells, and mossy fiber terminals (Fig. 3e, g).

At disease onset, the same timepoint at which RNA-seq was performed, we found that there were no significant differences in TMRE signals in Purkinje cells, as well as in molecular layer and granule cell layer from SCA6 and WT mice (Fig. 3d–e). This suggests that cellular respiration may be not affected at this age despite reduction in gene expression. However, since SCA6 is a progressive disease [27, 46], and disease alterations are thought to accumulate with disease progression, we also measured Δψ_m_ in SCA6 and WT mice at a later timepoint when disease had progressed (at 12 months, 5 months after disease onset). Strikingly, we observed a significant reduction in TMRE signals which indicated a reduction in mitochondrial Δψ_m_ in all cerebellar locations examined, including in Purkinje cell bodies, as well as in the molecular layer and granule cell layer in SCA6 cerebellar vermis compared to WT (Fig. 3f, g). While changes in the molecular layer could reflect alterations in mitochondria in both molecular layer interneurons and Purkinje cell dendrites, the alterations observed in the granule cell layer must arise from cell types other than Purkinje cells, which suggests that mitochondria dysfunction may not be limited to Purkinje cells in SCA6. We wondered if such dysfunction is limited to the cerebellum, or can also be observed in other brain regions. To investigate this, we measured TMRE signals in the somatomotor cortex from SCA6 and WT mice at the same disease timepoint (at 12 months; Supplementary Fig. 2). We oberved no significant difference in TMRE signals between the two genotypes (Supplementary Fig. 2b, c), while on the same tissue slice as an internal control, the TMRE signals of SCA6 Purkinje cells were significantly lower than that in WT (Supplementary Fig. 2d, e). This suggests that at this timepoint, mitochondrial dysfunction is restricted to the cerebellum, in line with the understanding that SCA6 pathology is largely cerebellar. The Δψ_m_ reduction we observed in SCA6 mice suggests that mitochondria have reduced cellular respiration, that is, a reduced capacity to produce ATP, as the disease progresses. Since Purkinje cells show high spontaneous activity [13], and thus require a high level of energy to sustain cellular function [11], the perturbation of ATP production may be particularly deleterious. Interestingly, since the transcriptional alterations we observed at disease onset were not reflected in functional changes until later during disease progression, our findings suggest that Purkinje cells normally have surplus bioenergetic capacity, making them resilient to small perturbations in mitochondrial gene expression.

### Purkinje cells are under oxidative stress as SCA6 progresses

**Fig. 4:**
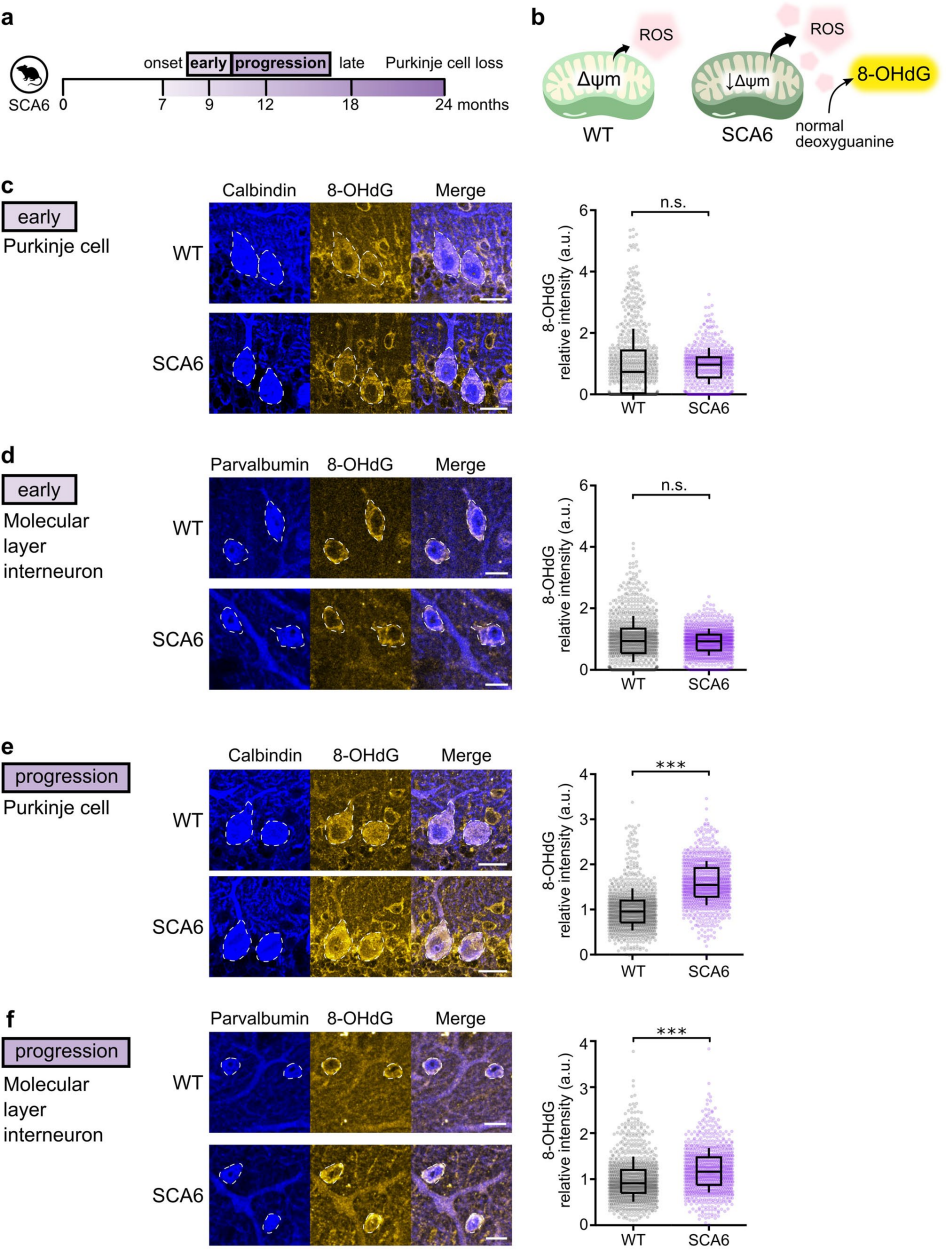
SCA6 cerebellar neurons accumulate oxidative stress as disease progresses. (**a**) Oxidative damage was measured at two disease timepoints: an early disease stage and disease progression stage. (**b**) Reduction in Δψ_m_ increases ROS production by mitochondria, which then oxidizes deoxyguanine to 8-OHdG. 8-OHdG is used as a biomarker for cellular oxidative stress (**c**, **d**) At an early disease stage, we did not observe a significant increase in oxidative stress (**c**) in SCA6 Purkinje cell bodies (WT: n = 695 cells from N = 3 WT mice; SCA6: n = 553 cells from N = 3 mice; P = 0.109) or (**d**) in molecular layer interneurons (WT and SCA6: n = 960 cells from N = 3 mice; P = 0.207). (**e**, **f**) At disease progression stage, we observed a significant accumulation of oxidative stress (**e**) in SCA6 Purkinje cells (WT: n = 952 cells from N = 3 WT mice; SCA6: n = 939 cells from N = 3 mice; P < 0.0001) and (**f**) in molecular layer interneurons (**f**) (WT: n = 960 cells from N = 3 WT mice; SCA6: n = 880 cells from N = 3 mice; P < 0.0001), a disease stage when Δψ_m_ was also significantly reduced. Scale bars for (**c**) and (**e**) = 20 µm; for (**d**) and (**f**) = 10 µm. Mann Whitney *U* test was used for all statistical comparisons. *** P < 0.0001, n.s. P > 0.05

Previous studies have shown that reduction in Δψ_m_ leads to increases in reactive oxygen species (ROS) production [65]. There is also evidence that damaged mitochondria can induce ROS production from other sources, such as the endoplasmic reticulum [47]. ROS accumulation is typically considered to be deleterious to cell health [74], and Purkinje cells have been shown to be particularly susceptible to oxidative stress [10]. Indeed, antioxidant treatments that reduce oxidative stress have been shown to prevent Purkinje cell death in mouse models of disease such as spinocerebellar ataxia type 1 (SCA1) and in Autosomal recessive spastic ataxia of Charlevoix-Saguenay (ARSACS) [43, 62]. Given that we observed a reduction in Δψ_m_ in the cerebellar vermis from SCA6 mice as disease progresses, we wondered if this would lead to excessive oxidative stress. To address this, we performed immunohistochemistry staining on acute cerebellar slices against a biomarker of cellular oxidative damage, 8-dihydro-2'-deoxyguanosine (8-OHdG), which is an oxidized form of the nucleotide guanine (Fig. 4b) [72]. We also labeled Purkinje cells with calbindin and interneurons with parvalbumin to localize these changes to specific cell types (Fig. 4c–f).

Since changes in Δψ_m_ were not detected at disease onset (Fig. 3), we chose to investigate two time points after onset: an early disease stage (9 months, 2 months after disease onset) and a later stage of disease progression (12 months; Fig. 4a). At the early disease stage, we observed robust labelling for the oxidative damage signal in both Purkinje cells as well as in molecular layer interneurons even in WT mice (Fig. 4c, d), which may represent oxidative damage related to normal aging. Staining in the granule cell layer was diffuse and not clearly localized to any cell type, so this was not quantified further. We observed no significant differences in the intensity of 8-OHdG in WT or SCA6 mice at this early disease stage (Fig. 4c, d), as expected given that we observed no significant changes in Δψ_m_ at disease onset (Fig. 3). However, as disease progressed, we observed a significant accumulation of the oxidative damage marker in both Purkinje cells bodies and molecular layer interneurons in SCA6 (Fig. 4e, f). This is also expected given the decrease in Δψ_m_ at the same progressive stage of disease (Fig. 3). Molecular layer interneurons have been classified into different subtypes [18] in part based on their location within the molecular layer of the cerebellum. To determine if a subtype of molecular layer interneurons was more susceptible to oxidative stress, we examined the upper- and lower- molecular layer interneuron subpopulations individually. We found that both populations had significantly elevated oxidative stress in SCA6 compared to WT (Supplementary Fig. 3). Taken together, the accumulation of oxidative stress we observe as SCA6 progresses suggests that mitochondrial dysfunctions could lead to broader negative consequences to cellular health.

### Mitochondria are damaged in SCA6 Purkinje cells

**Fig. 5:**
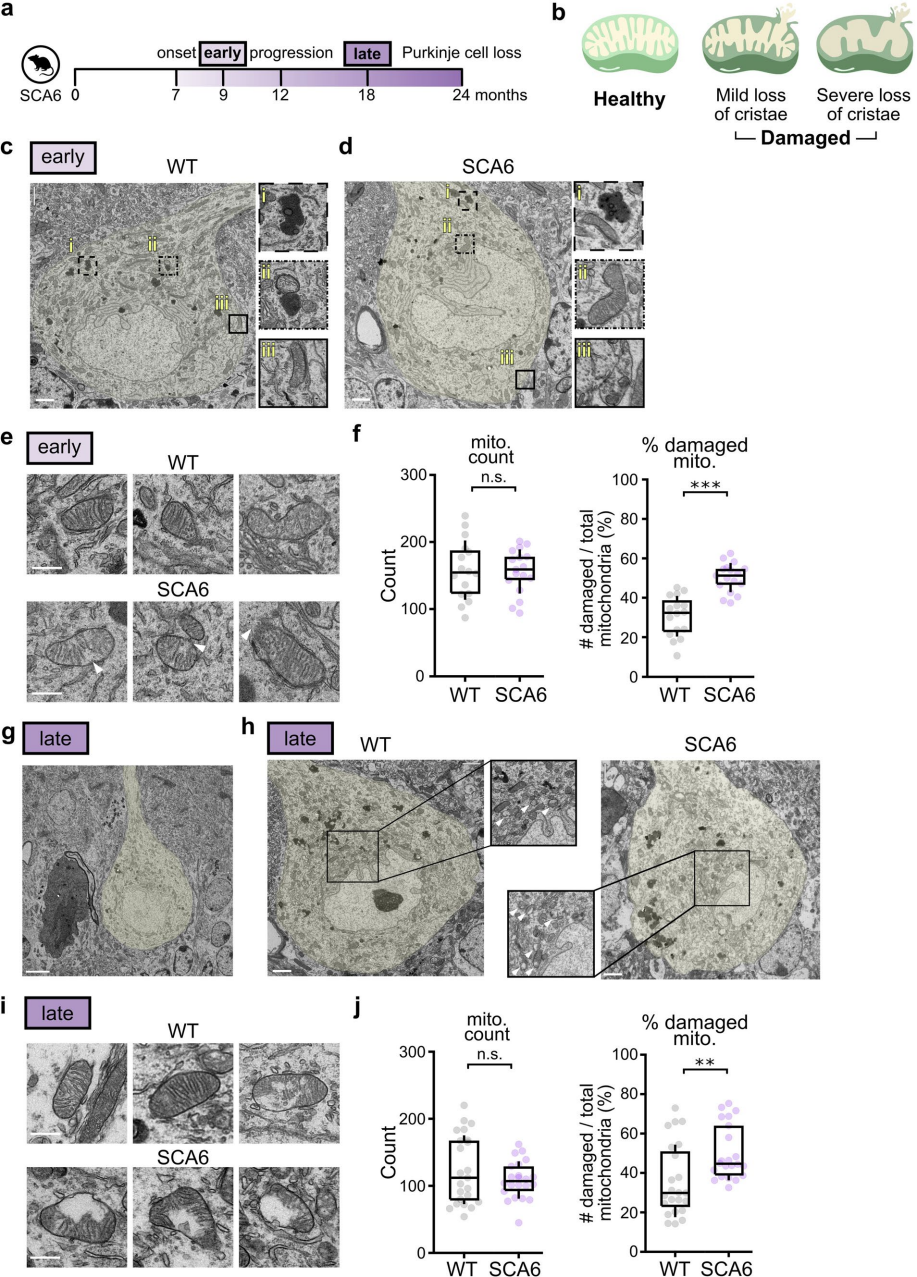
Ultrastructural features of WT and SCA6 Purkinje cell mitochondria. (**a**) The ultrastructure of mitochondria was studied at early and late disease time points. (**b**) Damaged mitochondria were identified by their loss of cristae, and/or ruptured mitochondrial membrane. (**c**, **d**) Representative images of WT and SCA6 Purkinje cells at early disease stage. (**e**) Representative images of mitochondria in WT and SCA6 Purkinje cells at early disease stage. White arrows show loss of cristae folding or rupture in mitochondrial membrane. (**f**) At early disease stage, we observed a similar number of mitochondria in both WT and SCA6 Purkinje cells (WT: 158 ± 11.1 mitochondria / cross-section of Purkinje cell body; SCA6: 156 ± 8.0 mitochondria / cross-section of Purkinje cell body; P > 0.05). There was a significantly higher proportion of damaged mitochondria in SCA6 Purkinje cells (WT: n = 16 Purkinje cell bodies from N = 3 mice, SCA6: n = 17 Purkinje cell bodies from N = 3 mice; P < 0.0001). (**g**) Dark cell degeneration of a Purkinje cell can be observed late in disease progression. (**h**) Representative images of Purkinje cells from WT and SCA6 cerebella. White arrows denote damaged mitochondria (**i**) Representative images of mitochondria in WT and SCA6 Purkinje cells at late disease stage. (**j**) At late disease stage, we observed similar number of mitochondria in both WT and SCA6 Purkinje cell (WT: 124 ± 10.7 mitochondria /cross-section of Purkinje cell body; SCA6: 109 ± 5.9 mitochondria/cross-section of Purkinje cell body; P > 0.05). There was a significantly higher proportion of damaged mitochondria in SCA6 Purkinje cells (WT: n = 23 Purkinje cell bodies from N = 3 mice, SCA6: n = 22 Purkinje cell bodies from N = 3 mice; P < 0.005). Scale bar in (**c**), (**d**) and (**h**) = 2 µm; in (**e**) and (**i**) = 0.5 µm; in (**g**) = 5 µm. Mann Whitney *U* test was used for all statistical comparisons. ** P < 0.005, *** P < 0.0001, n.s. P > 0.05

There is a close relationship between mitochondrial function and morphology. For example, disrupted mitochondrial cristae is a hallmark morphological feature that is tightly associated with impaired mitochondrial respiration [12]. Given the functional deficits we observed in SCA6 mice as disease progressed, we wondered if morphological changes in mitochondria would be evident in the SCA6 cerebellum. We used transmission electronic microscopy to examine the ultrastructure of mitochondria in SCA6 and WT Purkinje cell bodies from two disease stages: an early disease timepoint (9 months) and a late disease timepoint when disease progression has advanced (18 months; Fig. 5a). Purkinje cells can be visibly identified within a sagittal cut of the cerebellar vermis–(1) they are localized in a single-cell thick layer between the outer molecular layer filled with cross section cut parallel fibres and the inner granule cell layer populated by granule cells with visually-distinctive nuclei, and (2) Purkinje cell bodies are larger than other surrounding cell types (Supplementary Fig. 4a).

Ultrastructural examination of Purkinje cell bodies early in disease in WT and SCA6 mice revealed several hallmark features. (1) First, we observed lipofuscin granules (Fig. 5ci and di), an aging-related structure common to post-mitotic cells. Its formation has been linked to oxidative damage to proteins [51], mitochondrial impairment [34] and lysosomal dysfunction [64]. Interestingly, we observed no differences in the number of lipofuscin particles in WT and SCA6 Purkinje cells (Supplementary Fig. 4b), suggesting that its accumulation arises due to aging rather than disease. (2) Next, we observed putative autophagosomes (thick membranous structures enclosing disarrayed content) in proximity to a lysosome (dark, electron dense body) (Fig. 5cii). This association likely represents a snapshot showing the degradation of mitochondria and/or other cellular components. (3) We could also identify mitochondria-subsurface cisterna contact (Fig. 5ciii), and (4) mitochondria-endoplasmic reticulum (ER) contacts (Fig. 5dii). Both these last structures are thought to be enriched in Purkinje cells [20] and are involved in changes in calcium dynamics following synaptic stimulation [67]. Finally, we occasionally observed evidence of (5) synapses being made onto Purkinje cell bodies (Fig. 5diii).

At an early disease timepoint, we observed similar numbers of mitochondria in both WT and SCA6 Purkinje cells (Fig. 5f). We wondered whether morphological evidence for mitochondrial damage would be present at this early disease stage. Damaged mitochondria were identified as those with loss of cristae folding, and/or a rupture in the mitochondrial membrane (Fig. 5b). While damaged mitochondria were observed in WT Purkinje cells at this age, they were more common in SCA6 (Fig. 5e, f). This was surprising, since we lack functional evidence for mitochondrial impairment at this age (Figs. 3 and 4) and suggests that changes in morphology precede changes in function. We wondered if mitochondrial dysfunction worsens as disease progresses. To address this in more detail, we next examined mitochondrial morphology at a late stage of disease progression (18 months).

Notably, we observed dark cell degeneration at this late disease stage (Fig. 5g). Dark cell degeneration is a special form of cell death that Purkinje cells undergo, which has also been observed in other disease models that exhibit mitochondria dysfunctions [9, 39, 40]. Focusing only on Purkinje cell bodies which appeared healthy and did not display cytoplasmic darkening, we observed no significant difference in the number of mitochondria in WT and SCA6 Purkinje cells at this late stage of disease progression (Fig. 5h–j), similar to what we observed at an early stage of disease (Fig. 5f). However, the number of mitochondria in both genotypes decreased with age (compare Fig. 5f–j). We next examined damaged mitochondria in WT and SCA6 Purkinje cells, and found that the proportion of damaged mitochondria in SCA6 was elevated compared to in WT. Although the absolute proportion of damaged mitochondria was similar to the early disease timepoint, there are nonetheless strong signs of increased severity of damage with age in SCA6. For example, at 18 months the loss of cristae was more severe (compare Fig. 5i–e). Taken together, these data suggest that mitochondria display progressive morphological impairment, and that mild impairment precedes functional deficits in SCA6 mice in early stages of disease.

### Insufficient mitophagy in SCA6

The pool of mitochondria in a cell is maintained by a balance between mitochondria biogenesis, recycling through the fusion-fission process, and removal of the damaged mitochondria through mitophagy [42]. One puzzle in our data is that given the reduced expression of mitochondrial genes and the increase in damaged mitochondria with age, we might predict that we would observe a reduction in the total number of mitochondria. Yet this was not observed, suggesting that additional changes in mitochondrial removal may be present in the SCA6 cerebellum. To test this hypothesis, we took two experimental approaches. (1) quantification of lysosome and mitochondria colocalization by immunohistochemistry, and (2) quantification of Purkinje cell LC3 by immunohistochemistry.

**Fig. 6:**
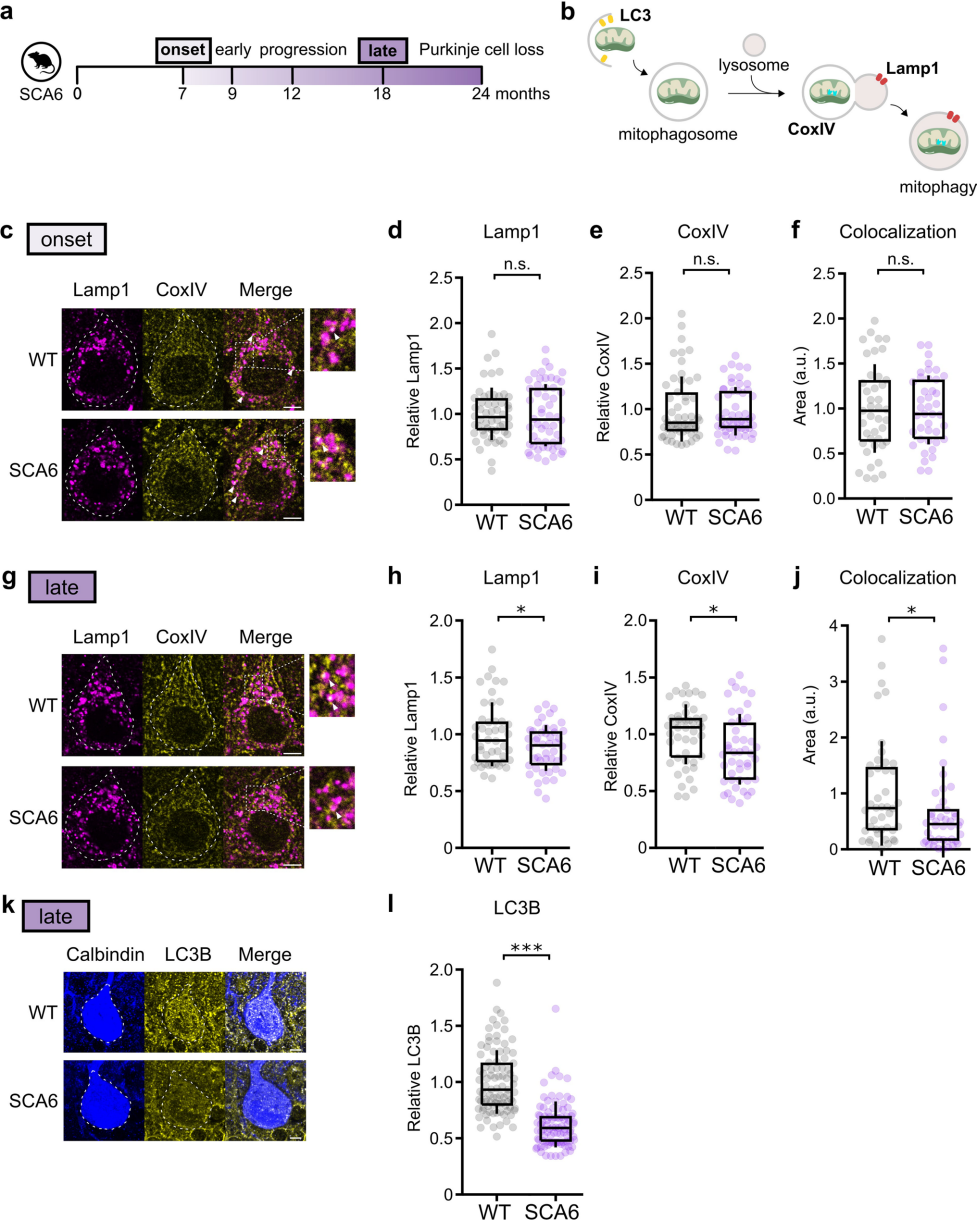
Insufficient mitophagy in SCA6 at late disease state. (**a**) Extent of mitophagy was studied at early and late disease stages. (**b**) Illustration showing autophagy marker LC3, mitochondria marker CoxIV and lysosome marker Lamp1 in the mitophagy pathway. (**c**) Representative images showing Lamp1 and CoxIV staining in Purkinje cell bodies (outlined) at early disease stage. Colocalization of the two markers can be observed (arrow heads). (**d**, **e**) Relative Lamp1 (**d**) and CoxIV (**e**) intensity within Purkinje cells were unchanged in SCA6 compared to WT controls (WT and SCA6: n = 54 cells from N = 3 mice; Student’s *t* test; P > 0.05). (**f**) The area of colocalization of Lamp1 and CoxIV staining within Purkinje cells was unchanged in SCA6 compared to WT controls (WT and SCA6: n = 41 cells from N = 3 mice; Student’s *t* test; P > 0.05). (**g**) Representative images showing Lamp1 and CoxIV staining in Purkinje cell bodies (outlined) at late disease stage. Zoomed in images shows that there was less colocalization in SCA6 Purkinje cells. (**h**, **i**) Relative Lamp1 (**h**) and CoxIV (**i**) intensity within Purkinje cells were reduced in SCA6 compared to WT controls (WT and SCA6: n = 46 cells from N = 3 mice; Student’s *t* test; P < 0.05). (**j**) The area of colocalization of Lamp1 and CoxIV staining within Purkinje cells was decreased in SCA6 compared to WT controls (WT and SCA6: n = 44 cells from N = 3 mice; Mann Whitney *U* test; P < 0.05). (**k**) Representative images showing Purkinje cell marker calbindin and autophagy marker LC3B staining in Purkinje cells at late disease stage. (**l**) Relative LC3B level within Purkinje cells was reduced in SCA6 compared to WT controls (WT and SCA6: n = 93 cells from N = 3 mice; Mann Whitney *U* test; P < 0.0001). Scale bars for all images = 5 µm. * P < 0.05, *** P < 0.0001, n.s. P > 0.05

The last step of the process of mitophagy is the fusion of mitochondria-containing autophagosomes with lysosomes (Fig. 6b). Therefore, the colocalization of mitochondria with lysosomes is a direct indication of mitophagy. To determine the level of mitophagy in SCA6 Purkinje cells, we quantified the expression levels and the degree of colocalization of the lysosome marker Lamp1 and mitochondria marker CoxIV in Purkinje cells by immunohistochemistry. At the age of disease onset, we observed no difference between WT and SCA6 animals in the fluorescence intensity or area of Lamp1 and CovIV within Purkinje cell bodies (Fig. 6c–e, Supplementary Fig. 4a, b), as well as no difference in the area of colocalization of these proteins (Fig. 6f). However, at a late disease stage, we observed reductions in both Lamp1 and CoxIV staining (Fig. 6g, i) as well as a significant reduction in their colocalization in SCA6 Purkinje cells compared to WT (Fig. 6j). This suggests that there is a reduction in mitophagy at late disease stages in cerebellar Purkinje cells in SCA6. Interestingly, the reduction in CoxIV intensity but not CoxIV area (Supplementary Fig. 5c, d) at this disease stage was consistent both with our finding in the transcriptomics data (Fig. 2b, c, Supplementary Table 1) as well as in our EM data (Fig. 5)–since CoxIV resides in the cristae of mitochondria, the loss of cristae with no change in mitochondria count is consistent with the reduction in intensity but not the total area of CoxIV immunoreactivity.

Second, we quantified the expression of the autophagosome marker MAP1 light chain 3 (LC3) since it is important for regulating autophagic flux and its expression level has been shown to be dysregulated in disease states that lead to insufficient autophagy (Fig. 6b) [56]. We co-stained acute cerebellar slices with an antibody against LC3B, an isoform of LC3, and the Purkinje cell marker calbindin. At a late disease stage, we observed a significantly reduced level of LC3B in Purkinje cell body compared to WT (Fig. 6k–l). Interestingly, we also observed reduced level of LC3B in the molecular layer and granule cell layer (Supplementary Fig. 5e, f). This suggests that autophagy may be impaired not only in SCA6 Purkinje cells but also in other cerebellar cell types at late disease stages. Taken together, these data suggest that in addition to the accumulation of increasingly damaged mitochondria in SCA6 mouse cerebellum, an impairment in mitochondrial removal via mitophagy contributes to mitochondrial dysfunction in SCA6.

### SCA6 patient’s cerebellar tissues showed metabolic signature for mitochondrial dysfunction

One open question with these findings is whether the results from SCA6 mice are representative of human disease. To address this, we acquired post-mortem cerebellar tissues from SCA6 patients and age- and sex- matched non-neuropathological individuals (Supplementary Table 3) to determine whether signatures of mitochondrial dysfunction were present in SCA6 post-mortem cerebellum.

**Fig. 7:**
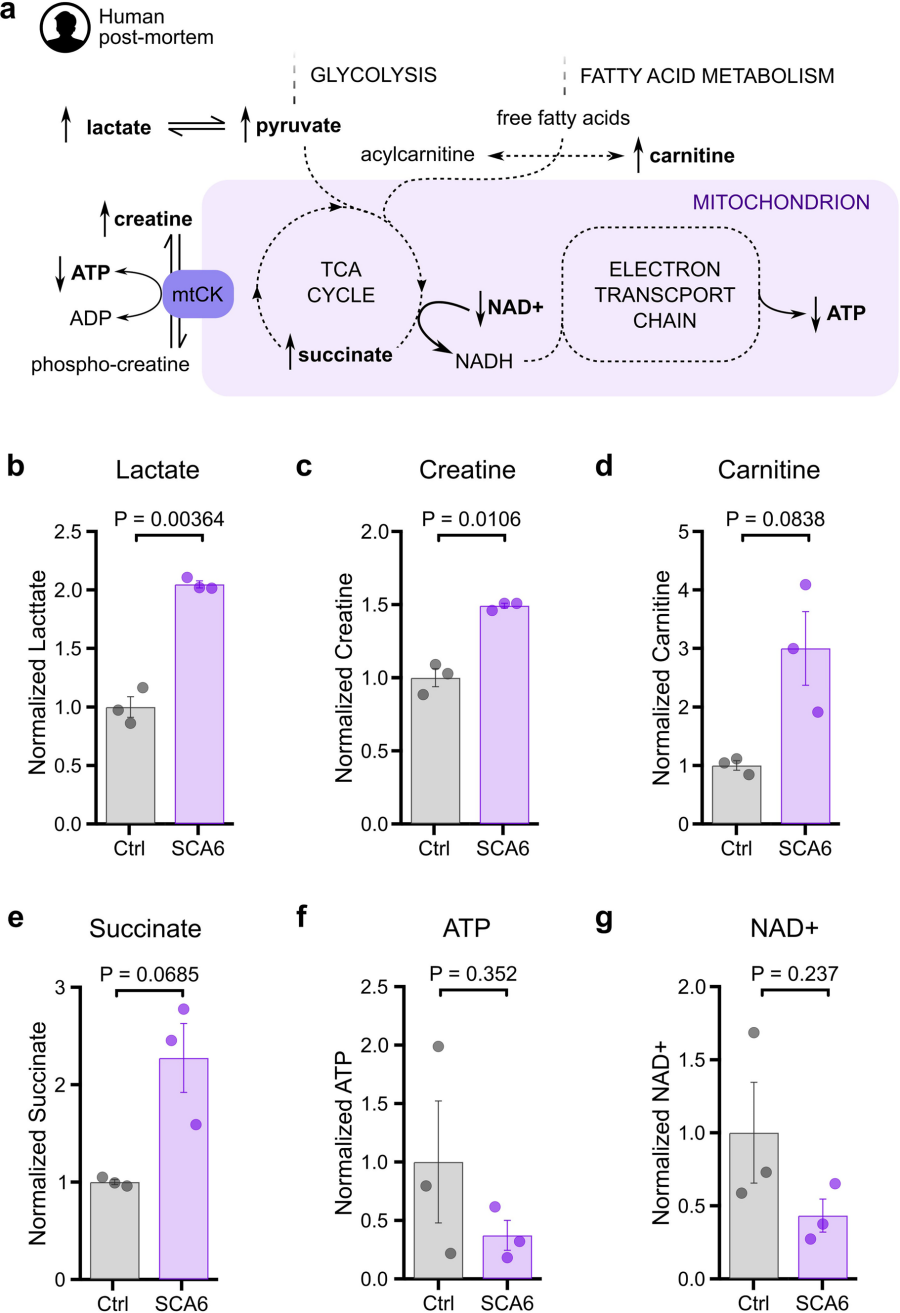
Biomarkers for mitochondrial dysfunctions were detected in SCA6 patient’s cerebellar tissues. (**a**) Simplified illustration of energy metabolism pathways, highlighting biomarkers for mitochondrial dysfunctions (bolded) and their predicted direction of change based on previous literature. Levels of lactate (**b**) and creatine (**c**) were significantly increased in SCA6 patients’ tissues, suggesting mitochondrial oxidative phosphorylation failure. There were non-significant but trending changes in the predicted direction detected in carnitine (**d**) and succinate (**e**). ATP (f) and NAD + (**g**) changes were non-significant but were consistent with predicted changes under mitochondrial dysfunctions. Unpaired Student’s *t* test was used for all statistical comparisons. Ctrl: Non-neuropathological controls

To examine this question, we used liquid chromatography-mass spectrometry (LC/MS) to analyze biomarkers from human post-mortem cerebellar tissue to look for evidence of mitochondrial dysfunctions and elevated oxidative stress (Fig. 7a). Classic biomarkers for mitochondrial dysfunction include lactate, pyruvate, creatine, and carnitine, which are alternate energy metabolites that can be important for high-energy-demanding tissue like the brain. Previous work has shown that brain levels of these biomarkers increase in response to oxidative phosphorylation dysfunction [24, 68, 73]. We also expected to observe the accumulation of succinate as a result of reduced enzymatic activity of succinate dehydrogenase, or ETC complex II, an important bioenergetic enzyme that functions both in the Krebs cycle and the ETC [7]; a reduction of ATP production and a disequilibrium of the ETC cofactors NAD + /NADH [69, 77].

We were able to detect many molecules relevant for mitochondrial function in SCA6 post-mortem cerebellar tissues, which we compared to control tissues (Supplementary Table 4). Overall, our findings support mitochondrial dysfunction in the cerebellum of SCA6 patients. First, we observed a significant two-fold increase in the level of lactate (Fig. 7b). When mitochondria are damaged and the ETC fails, cells shift their energy production from mitochondrial oxidative phosphorylation towards the less efficient glycolysis, generating excess lactate as a result [36]. We also observed a significant increase in creatine (Fig. 7c), and a non-significant increase in carnitine (Fig. 7d). These features together suggest that oxidative phosphorylation dysfunction is observed in the human SCA6 cerebellum.

Furthermore, we found a non-significant but trending increase of succinate in SCA6 cerebellum (Fig. 7e), which has been shown to induce higher cellular oxidative stress and mitochondria dysfunctions [31, 38, 79]. Both ATP and NAD + are known to be decreased with mitochondrial dysfunction and in mitochondrial diseases [8, 44, 61]. We thus quantified the level of ATP and NAD + and found that both were present at a lower, though not statistically significant, level in SCA6 patients’ cerebellum (Fig. 7f, g). To summarize, all biomarker changes we observed were in the direction predicted by mitochondrial dysfunction, although not all changes were significantly altered. It is noteworthy that some molecules are prone to undergo post-mortem changes, and to minimize the impact, we matched the post-mortem intervals (PMI) of control tissues with that of the SCA6 tissues (Supplementary Fig. 6a). For example, we observed variable levels of creatinine across individual samples, a metabolite that is commonly used in forensic analysis to estimate the PMI, which likely represent post-mortem changes and storage condition differences (Supplementary Fig. 6b) [3, 23]. However, some metabolites are known to be relatively stable despite long PMI, such as lactate [58], the biomarker that showed the most significant changes in our post-mortem SCA6 tissue. Taken together, the metabolic changes we observed in SCA6 patients’ tissues are consistent with mitochondrial impairment, supporting our findings from our SCA6 mouse model.

## Discussion

**Fig. 8:**
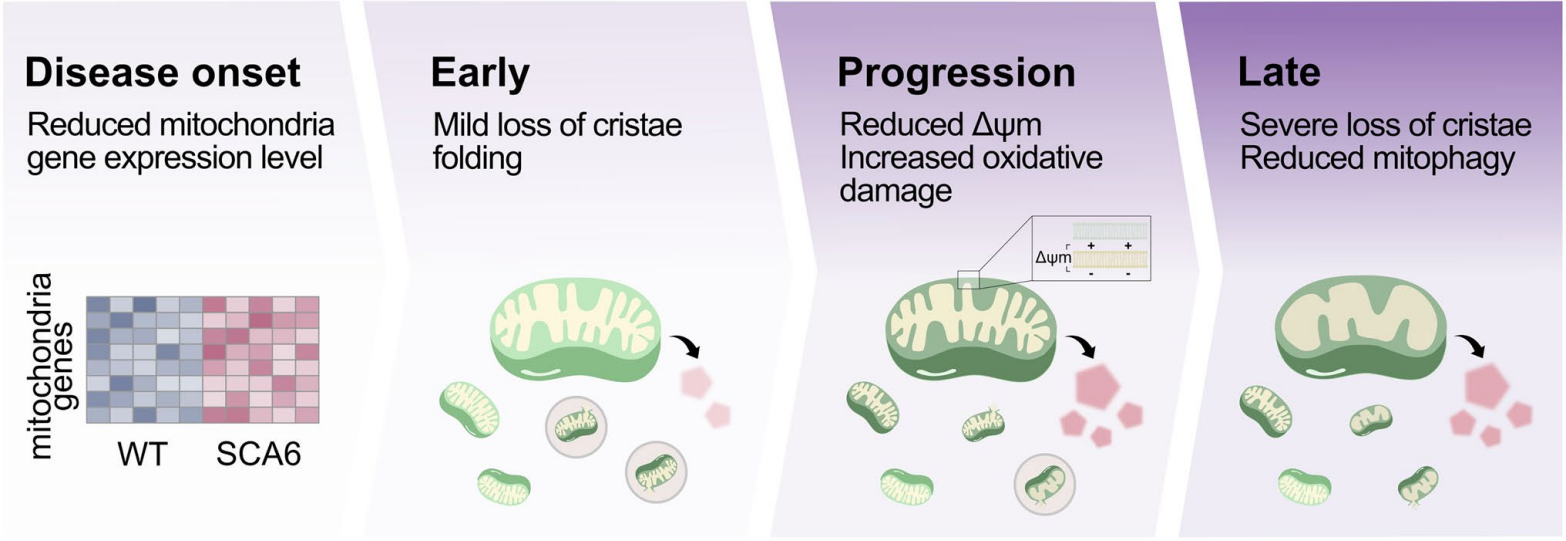
Mitochondrial dysfunction and structural impairment worsen over the course of SCA6 disease progression

Here we use multiple approaches to identify mitochondrial dysfunction as an important and hitherto unappreciated feature of SCA6 disease progression (Fig. 8). Bioinformatic analysis of transcriptomics data from disease onset revealed that mitochondrial-related genes were the most dysregulated gene families. Intriguingly, although transcriptional alterations were present at disease onset, a significant reduction in Δψ_m_ and a downstream increase in oxidative stress were not observed until later during disease progression, meaning that functional changes lagged behind transcriptional changes by several months. Structurally, Purkinje cell mitochondria were damaged both early and at a late stage of disease progression, with the degree of damage increasing with age. This accumulation of mitochondria with increasing structural damage appears to arise at least in part due to insufficient mitophagy at late disease stages, which will exacerbate mitochondrial impairment over time. Metabolomic analysis of SCA6 post-mortem cerebellar tissues revealed metabolic signatures of mitochondrial dysfunctions consistent with the findings from our mouse model.

Our data argue that mitochondrial dysfunction is an important feature of SCA6, yet it is one that has been previously unappreciated. However, fatigue, which is a hallmark feature of mitochondrial disease [21], is prevalent in SCA6 patients [4], and is often characterized as one of the worst symptoms of their disease by patients [4]. In fact, fatigue is a common comorbidity of many neurodegenerative diseases, and can describe both mental and/or physical exhaustion [48]. Recent work in human subjects has demonstrated a role for cerebellar activity in the perception of fatigue [6]. Perception of fatigue is difficult to study in animal models but is linked to the mental exhaustion associated with fatigue [2]. This work suggests a link may exist between fatigue and cerebellar function. Future studies will be needed to determine whether a link between mitochondrial impairment in the cerebellum and fatigue exists in SCA6.

One of the surprises from our data is the difference in the timelines we observed between transcriptional, structural, and functional changes related to mitochondria. Transcriptional changes occurred first at disease onset, as did mild alterations in Purkinje cell mitochondrial structure. However, functional changes or resulting alterations in oxidative stress were not observed at this time. It was not until much later during disease progression that mitochondrial dysfunction was observed, as well as a worsening of structural impairment. This shows that cerebellar neurons can maintain mitochondrial function even in the face of mild impairments, arguing that cerebellar neurons normally function with a large reserve of mitochondrial capacity.

Impaired Purkinje cell firing is a common feature of ataxias and other diseases affecting the cerebellum [13]. We have previously characterized altered Purkinje cell firing in SCA6 [14, 28]. Recent work has suggested a link between mitochondria and the regulation of neuronal firing [53, 63]. Thus, impaired mitochondrial respiration in SCA6 may contribute to Purkinje cell firing deficits at late stages of disease [14, 28], although other mechanisms, including mitochondrial Ca^2+^ buffering capacity, are likely to contribute to early impairment of firing rates.

Although a great deal of research in SCA6 has focused on Purkinje cells, which express the mutated protein at high levels and show late degeneration in mice [29] and patients [25], one notable discovery in our data is that mitochondrial impairment is not limited to cerebellar Purkinje cells but extends to other major classes of cells including molecular layer interneurons and granule cells. This finding is in line with recent studies showing the involvement of other cell classes that contribute to disease in other forms of ataxia, such as molecular layer interneurons in SCA1 [49]. Whether changes to other cells occur in parallel with changes in Purkinje cells or arise as a consequence of changes in Purkinje cells, remains to be determined. However, the mutated *CACNA1A* gene is expressed not only in Purkinje cells, but also in cerebellar granule cells [16, 19] and our in silico cell-type analysis of our transcriptomics data reveals that altered genes in SCA6 are likely expressed in many cell types (Supplementary Fig. 1e).

We propose the accumulation of damaged mitochondria is due to insufficient mitophagy, which is in line with evidence from our lab that there are endo-lysosomal deficits in SCA6 [15]. Such deficits could lead to reduced quality control mechanisms for mitochondria that contribute to Purkinje cell deficits and eventual cell death. The combination of mitochondrial deficits, oxidative damage, and reduced mitophagy suggests a deleterious cycle that increases the pathophysiological stress on Purkinje cells and other cerebellar cell types as disease progresses.

Many SCA6 patients are not diagnosed until well past disease onset, meaning that it is imperative to study mechanisms that contribute to disease progression as well as onset. Previous work in our lab shows that around disease onset, BDNF-TrkB signaling contributes to SCA6, and that activating this signaling pathway can reverse ataxia at early disease stages [14]. However, we found that as disease progresses, BDNF-TrkB signaling is no longer sufficient to reverse ataxia, suggesting that other mechanisms likely contribute to SCA6 as disease progresses. Mitochondrial dysfunction aligns with the timeline of disease progression: it worsens as the disease worsens. Mitochondrial function is important for the health of Purkinje cells; thus we propose that the mitochondrial dysfunction we describe here is likely to contribute to disease progression. This is important clinically because much biomedical research focuses on disease onset, but mechanisms that contribute to disease onset may not be appropriate therapeutic targets at late disease stages.

While SCA6 mice have been shown to recapitulate key properties of human disease [29, 76], it can be difficult to determine whether pathophysiological changes identified in animal models are representative of human disease. Even for a given mutation in human patients, disease presentation can vary widely. For example, in repeat expansion disorders such as SCA6, different repeat lengths can lead to different disease symptoms and pathology [66]. To tie these results more closely with human disease, we used metabolomics to determine whether signatures of mitochondrial deficits exist from post-mortem human tissue, representing a very late stage of disease progression. Our findings are consistent with deficits in mitochondrial function in human post-mortem cerebellar vermis, which validates findings from our mouse model. Taken together, findings from both mouse and human data argue that mitochondrial dysfunction is an important deficit that likely contributes increasingly to cerebellar damage as SCA6 progresses. How the CAG expansion mutation leads to these changes remains to be determined, although alterations in mitochondrial genes expression due to abnormal activity of the α1ACT transcription factor are likely to be involved [17]. Furthermore, our study suggests that by implementing therapeutic approaches to improve mitochondria health–for example, by inducing mitophagy or mitochondrial biogenesis [45, 71] –we could improve cerebellar cell health and function. This could lead to improvement in the progression or severity of ataxia in SCA6 [43] in particular at late disease stages that are unlikely to be ameliorated by other therapeutic approaches that target mechanisms of disease onset.

### Supplementary Information

Below is the link to the electronic supplementary material.

Supplementary file1 (xlsx 70 KB)

Supplementary file1 (pdf 2576 KB)

## Declarations

### Data availability

The raw sequence data reported in this paper have been deposited in the Genome Sequence Archive (accession: CRA013247) that are publicly accessible at https://ngdc.cncb.ac.cn/gsa.
