# Supplementary material for: Mitochondrial damage and impaired mitophagy contribute to disease progression in SCA6
Source: Acta Neuropathol. 2024 Jan 29;147(1):26. doi: 10.1007/s00401-023-02680-z (PMC10824820; doi:10.1007/s00401-023-02680-z)
Supplement: Supplementary file 2 — Supplementary file1 (pdf 2576 KB) [file 401_2023_2680_MOESM2_ESM.pdf]

Mitochondrial damage and impaired mitophagy contribute to disease progression in SCA6

Supplementary Information

Tsz Chui Sophia Leung[1], Eviatar Fields[1,2], Namrata Rana[1], Ru Yi Louisa Shen[1], Alexandra E. Bernstein[1], Anna A. Cook[1], Daniel E. Phillips[1], Alanna J. Watt[1*]

[1]Biology Department, McGill University, Montreal, QC, Canada

[2]Integrated Neuroscience Program, McGill University, Montreal, QC, Canada

[*]Correspondence: alanna.watt@mcgill.ca

Supplementary Information

Supplementary Figures 1-6

Supplementary Table 1 List of DEGs

Differentially expressed genes in SCA6 mice compared to WT controls, their fold changes and p values.

Supplementary Table 2 List of reagents and antibodies

Information of reagents and antibodies used in the current study and concentrations used.

Supplementary Table 3 Human post-mortem tissues demographics

Age, sex, post-mortem delay, CAG repeat length (SCA6 patients only) and sources of post-mortem human tissues.

Supplementary Table 4 LC/MS Raw data

Raw data and normalization factors for LC/MS datasets: amino acids and ion-pairing

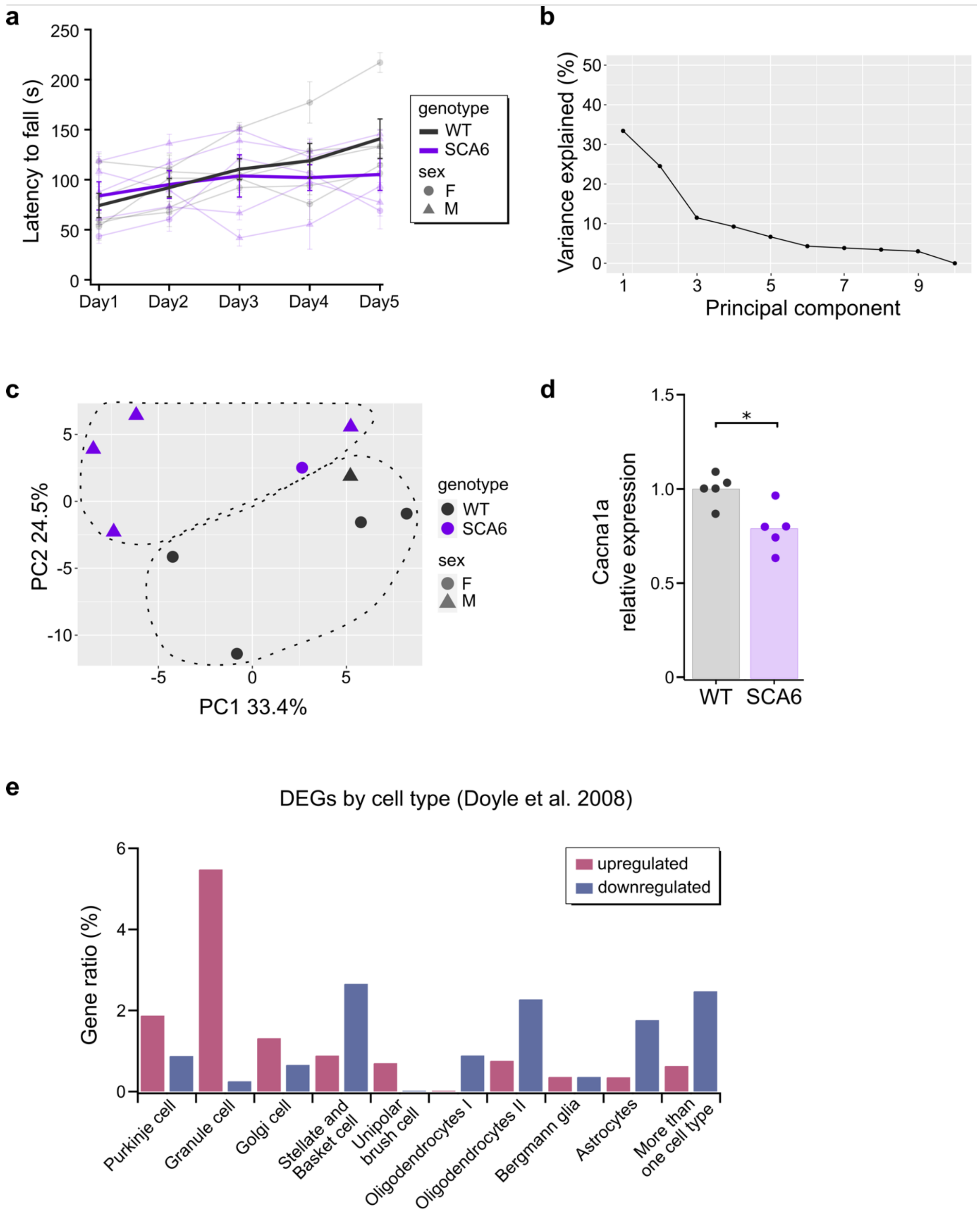

Supplementary Figure 1 (a) Motor coordination deficit of mice used for RNA-seq was assayed on rotarod. (b) Percentage of variance explained by each principal component (PC). (c) PC1 and PC2 together explain more than 50% of the variance, and animals of each genotype loosely cluster together (grouped with dashed line). (d) Relative expression level of *Cacna1a* in SCA6 is 20% lower than in WT (e) DEGs identified are specific for different cell types

in the cerebellum. Gene ratios are defined as the number mapped DEGs to a cell type / the number of cell-specific genes to that cell type identified in (Doyle et al. 2008).

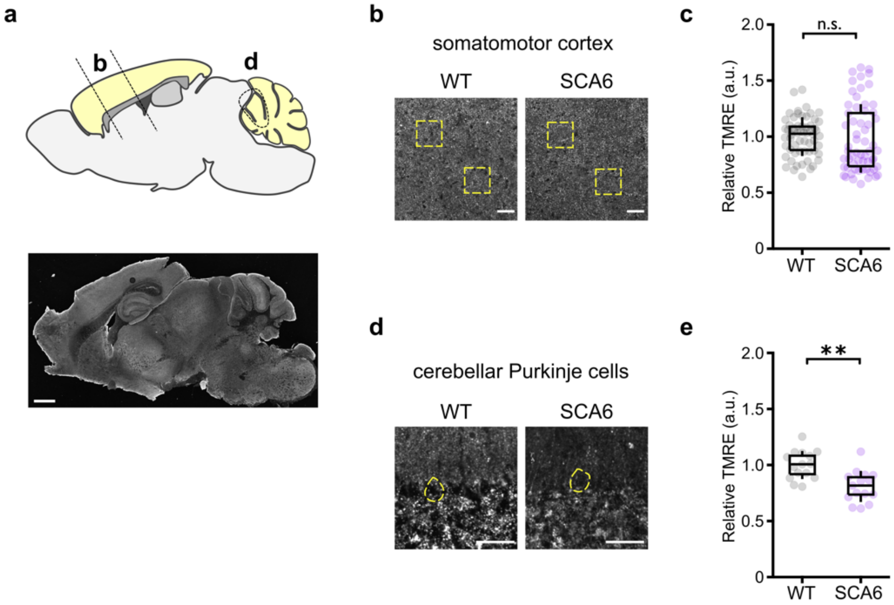

Supplementary Figure 2 Mitochondrial dysfunction was not detected in somatomotor cortex. (a) Top: Illustration showing regions where TMRE signals were measured from (regions within dashed lines). The somatomotor cortex is located using the corpus callosum, lateral ventricle and hippocampus as landmarks. Bottom: representative whole brain sagittal slice stained with TMRE. Image was taken at low magnification for reference purpose only. Analysis was performed on images taken on higher magnifications. (b) Representative TMRE images of somatomotor cortex in WT and SCA6 mice. (c) Relative TMRE signals showed no difference between genotypes (WT: n = 60 ROIs from N = 3 mice; SCA6: n = 60 ROIs from N = 3 mice; not significantly different, P = 0.724) (d) Representative TMRE images of Purkinje cells (outlined). (e) TMRE signals were significantly reduced in SCA6 (WT: n = 15 cells from N = 3 mice; SCA6: n = 15 cells from N = 3 mice, P = 0.000473). Scale bar for (a) = 100 µm, (b) and (d) = 50 µm. Mann Whitney *U* test was used for all statistical comparisons. ** P < 0.005, n.s. P > 0.05

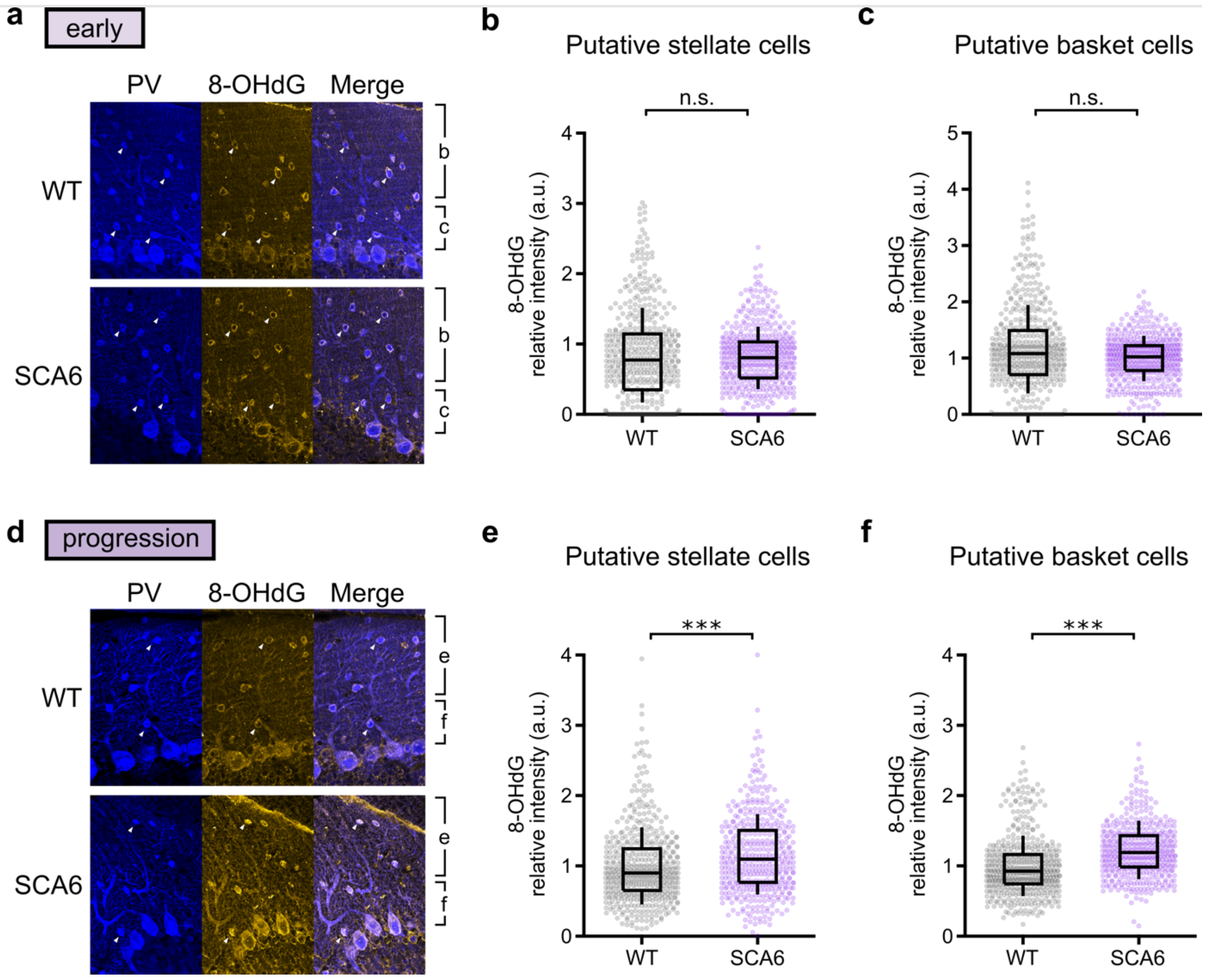

Supplementary Figure 3 Oxidative stress accumulates in both populations of molecular layer interneurons. (a) Representative images of molecular layer interneurons (stained with parvalbumin, PV) and oxidative stress marker (stained with 8-OHdG) at early disease stage. White arrows denote individual interneurons. The upper interneurons are putative stellate cells; the bottom interneurons are putative basket cells. Quantifications of oxidative stress staining of these two populations of interneurons are shown in (b) and (c) respectively. (b-c) Neither population shows significant difference in oxidative stress level compared to WT. (WT and SCA6: n = 480 cells from N = 3 mice; Mann Whitney *U* test, P > 0.05) (d) Representative images of molecular layer interneurons and oxidative stress staining at disease progression stage. (e-f) Both putative stellate cells and putative basket cells accumulated higher level of oxidative stress compared to WT. (WT and SCA6: n = 480 cells from N = 3 mice; Mann Whitney *U* test, P < 0.0001). *** P < 0.0001, n.s. P > 0.05.

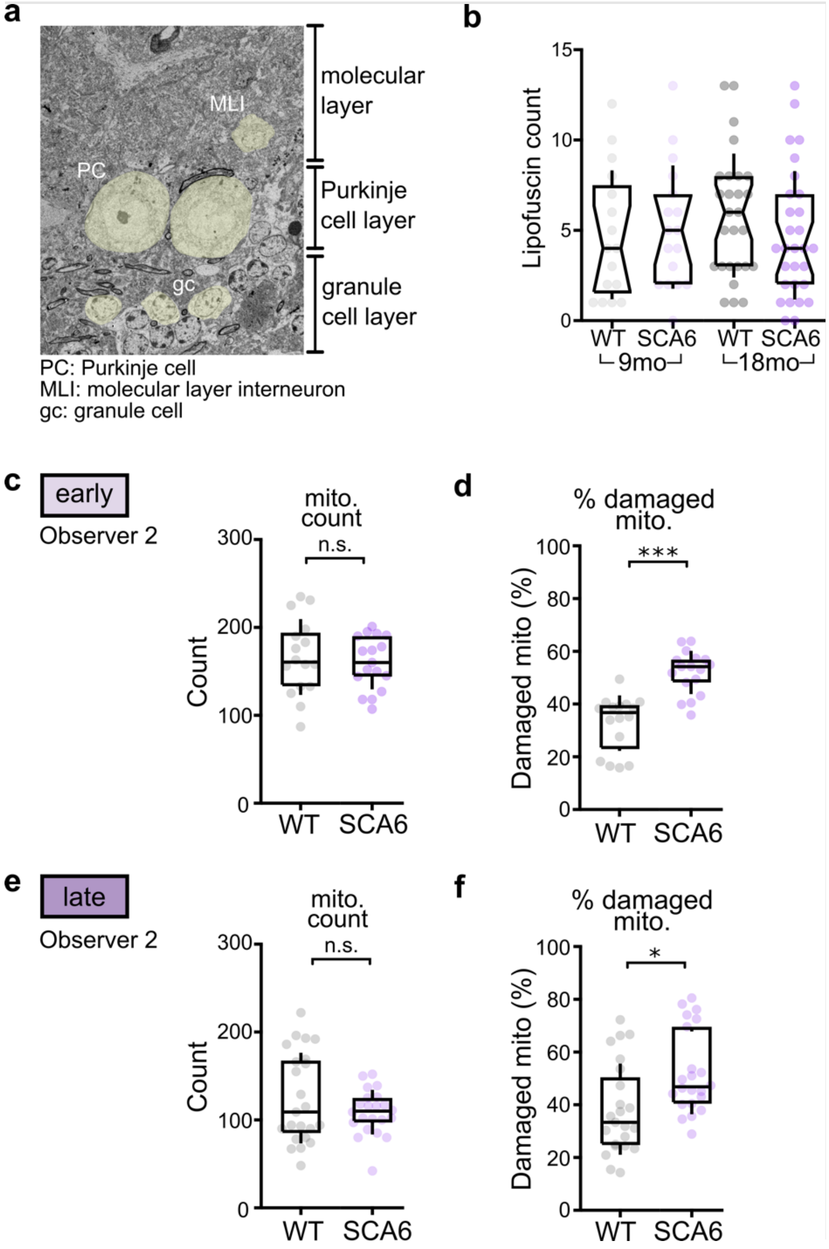

Supplementary Figure 4 (a) Location of Purkinje cells under electron microscope can be identified between the molecular layer interneurons and granule cells, which are both smaller. (b) Quantification of lipofuscin granules in Purkinje cell bodies. (c-d) Mitochondria count and damaged mitochondria percentage at early disease stage as quantified by observer 2. (e-f) Mitochondria count and damaged mitochondria percentage at late disease stage as quantified by observer 2. * $P < 0.05$, *** $P < 0.0001$, n.s. $P > 0.05$.

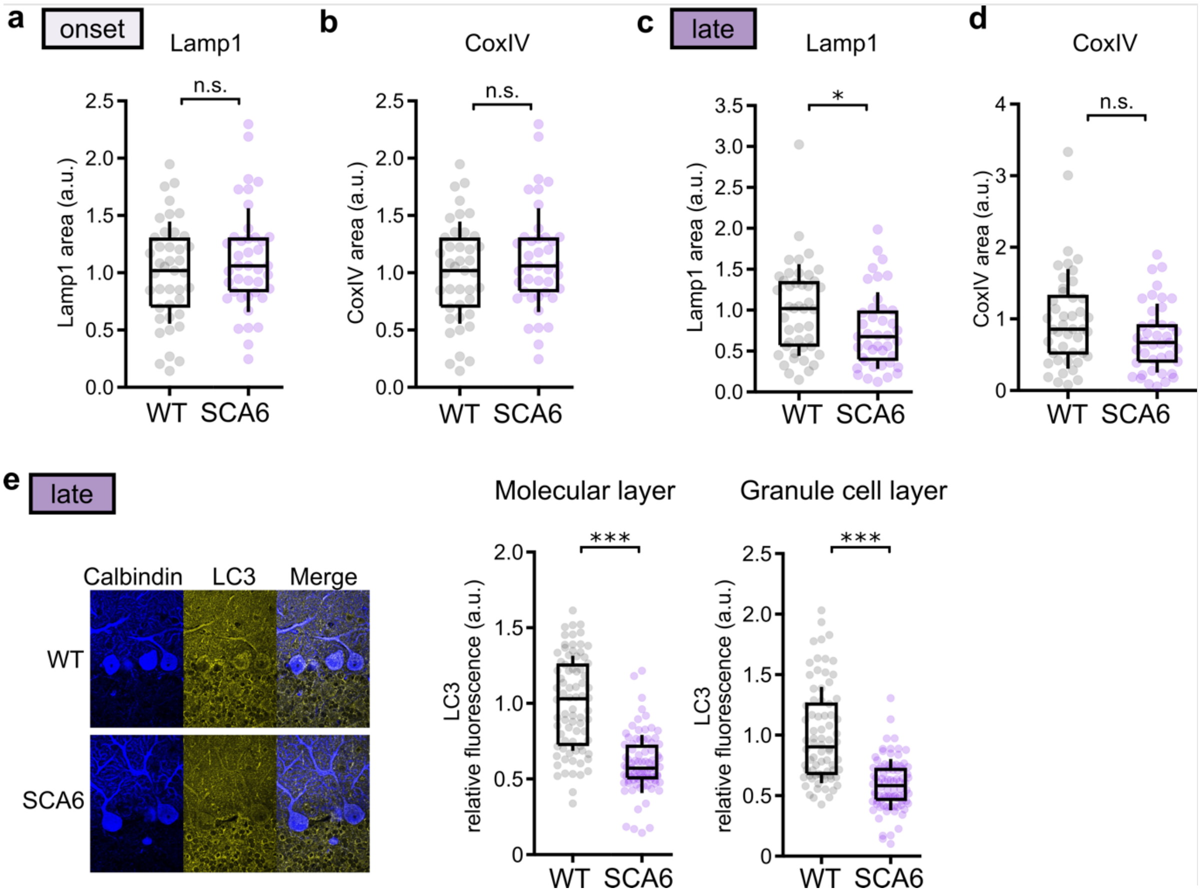

Supplementary Figure 5 (a-b) Lamp1 and CoxIV staining areas in Purkinje cell bodies are not altered in SCA6 at disease onset (c) Lamp1 area is reduced in Purkinje cell bodies in SCA6 at late disease stage, which is in line with the reduced fluorescence density. (d) CoxIV area is unchanged in Purkinje cell bodies in SCA6 at late disease stage, consistent with the EM data that there was no difference in mitochondria count between SCA6 and WT Purkinje cells at this disease stage. (e) LC3 fluorescence signals are reduced in both molecular layer and granule cell layer. * P < 0.05, *** P < 0.0001, n.s. P > 0.05.

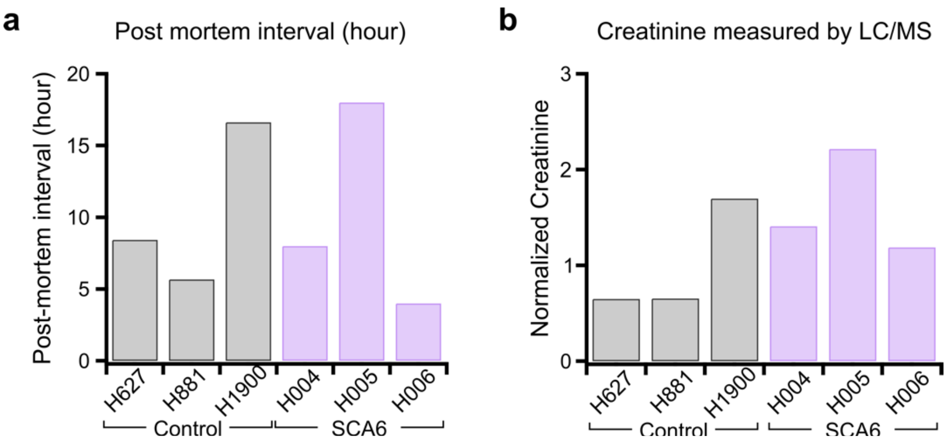

Supplementary Figure 6 Post-mortem delay of human post-mortem tissues (a) Post-mortem delay (hour) of control and SCA6 cerebellar tissues are matched to control for impact on metabolites cause by post-mortem delay. (b) Relative creatinine level in post-mortem human tissues used as an estimate for post-mortem-delay-related metabolic alterations.
